# Body condition influences ontogeny of foraging behavior in juvenile southern elephant seals

**DOI:** 10.1002/ece3.4717

**Published:** 2018-12-18

**Authors:** Florian Orgeret, Sam L. Cox, Henri Weimerskirch, Christophe Guinet

**Affiliations:** ^1^ Centre d’Etudes Biologique de Chizé UMR 7372 ‐ CNRS & Université de La Rochelle Villiers‐en‐Bois France; ^2^ Centre National d'Études Spatiales (CNES) 18 Avenue Edouard Belin 31400 Toulouse France; ^3^ MARBEC (Institut de Recherche pour le Developpemente; IRD) Station Ifremer de Sete, Avenue Jean Monnet, CS 30171, 34203 Sète France

**Keywords:** accelerometers, diving behavior, early‐life, first‐year juveniles, foraging behavior, ontogeny, satellite relay tags, southern elephant seals

## Abstract

Ontogeny of diving and foraging behavior in marine top predators is poorly understood despite its importance in population recruitment. This lack of knowledge is partly due to the difficulties of monitoring juveniles in the wild, which is linked to high mortality early in life. Pinnipeds are good models for studying the development of foraging behaviors because juveniles are large enough to robustly carry tracking devices for many months. Moreover, parental assistance is absent after a juvenile departs for its first foraging trip, minimizing confounding effects of parental input on the development of foraging skills. In this study, we tracked 20 newly weaned juvenile southern elephant seals from Kerguelen Islands for up to 338 days during their first trip at sea following weaning. We used a new generation of satellite relay tags, which allow for the transmission of dive, accelerometer, and location data. We also monitored, at the same time, nine adult females from the colony during their post‐breeding trips, in order to compare diving and foraging behaviors. Juveniles showed a gradual improvement through time in their foraging skills. Like adults females, they remarkably adjusted their swimming effort according to temporal changes in buoyancy (i.e., a proxy of their body condition). They also did not appear to exceed their aerobic physiological diving limits, although dives were constrained by their smaller size compared to adults. Changes in buoyancy appeared to also influence their decision to either keep foraging or return to land, alongside the duration of their haul outs and choice of foraging habitat (oceanic vs. plateau). Further studies are thus needed to better understand how patterns in juveniles survival, and therefore elephant seal populations, might be affected by their changes in foraging skills and changes in their environmental conditions.

## 1 INTRODUCTION

Animal behavior is influenced by evolutionary mechanisms, such as adaptation and phylogeny, and proximate mechanisms, such as physiology and ontogeny (“the four Tinbergen questions,” Tinbergen, 1963). In aquatic environments, dives performed by air‐breathing animals are constrained by a need to return to the surface to replenish oxygen supplies (Butler & Jones, 1997). Diving behavior, in this case, provides a good example of how a combination of the four Tinbergen questions can be applied to understand and describe characteristics of complex behaviors (Bateson & Laland, 2013). In spite of the variety of adaptations that exist (Ponganis, 2015), in marine bird and mammal species, the constraint imposed by foraging underwater is a powerful selective force (the first question of “adaptation”), which leads to a high degree of evolutionary convergence (the second question of “phylogeny”; Halsey, Butler, Blackburn, Huey, & Losos, 2006). A large number of studies have described the influence of physiology (the third question) on the ability of an individual to store oxygen, which in turn affects their diving aptitude (Costa, Kuhn, Weise, Shaffer, & Arnould, 2004). However, relatively few studies have investigated the fourth Tinbergen question: the importance of ontogeny on diving behavior in juvenile marine air‐breathing predators (Grecian, Lane, Michelot, Wade, & Hamer, 2018; Horning & Trillmich, 1997). This is likely because tracking juveniles for an appropriate period of time is challenging, particularly since this life stage typically displays high mortality rates (Hazen et al., 2012).

Several factors may influence the ontogeny of diving behavior of juvenile marine air‐breathing predators. This includes physiological development, such as growth, size, body composition, and apnea capacity (Kooyman, 1989), alongside experience acquisition such as cognition, memory development, and learning ability (Dukas & Ratcliffe, 2009). Environmental factors, such as aspects of the physical seascape, including biophysical oceanographic features, may also influence the diving behavior of individuals (Austin, Bowen, McMillan, & Iverson, 2006; Halsey, Blackburn, & Butler, 2006), by impacting prey availability abundance and distribution (Charrassin, Le Maho, & Bost, 2002). The ability of juveniles to locate high‐quality foraging patches in their environment is probably less developed or nonexistent compared to adults, putting them at a competitive disadvantage (de Grissac, Bartumeus, Cox, & Weimerskirch, 2017), such that they need to rapidly learn. If juveniles fail to catch prey at a rate high enough to survive, they will need to compensate by increasing their foraging effort (Daunt, Afanasyev, Adam, Croxall, & Wanless, 2007). If this increase is beyond their physiological capacity they may die (Daunt et al., 2007; Orgeret, Weimerskirch, & Bost, 2016).

In theory, the physiological limits of a diver correspond to the threshold dive duration when lactate concentration starts to be accumulated (the Aerobic Dive Limit, ADL, Kooyman, 1989). However, lactate measurements in the field are difficult to perform, and other methods are often used which measure the oxygen stored by the animal divided by the estimated consumption rate of oxygen when the animal is diving (the theoretical ADL, tADL). With an animal that lives at sea for several months, obtaining these two measurements (lactate or oxygen) are not possible, and thus a third method has been developed that relies on the diving behavior of the animal (the behavioral ADL, bADL). This limit corresponds to dive duration where the next surface duration starts to increase exponentially. Even if a sharp increase in surface duration would not be necessarily associated with a switch to anaerobic metabolism, this bADL is often considered to be a proxy of a physiological limit and an indicator of an increase in diving effort (Kooyman, 1989). As such, the development of foraging aptitude in juveniles has a strong influence on their life history and fitness (Stearns, 1976), alongside the demographic trends of populations (Sæther et al., 2013). To survive, young animals must be able to improve their foraging skills by learning as quickly as possible how to find and capture their prey, and how to adjust their foraging effort without exceeding their physiological limits (Ponganis, 2015).

Recent developments in pinniped bio‐logging technologies allow the measurement and transmission of both behavioral and physiological information (e.g., prey capture rates; PrCA), swimming effort and changes in buoyancy from accelerometer and depth measurements, Cox et al., 2017). Pinnipeds represent good models for studying the development of foraging strategies because juveniles are large and can robustly carry electronic devices for many months (Carter et al., 2017). Most seals become nutritionally independent from their mothers before foraging independently, so parental influence on foraging skill development is considered to be null after independence. Condition at departure is thus largely influenced by feeding prior to weaning and should strongly influence the amount of time available to develop foraging skills and therefore the probability of survival (McMahon, New, Fairley, Hindell, & Burton, 2015).

In deep‐diving phocids like elephant seals, the buoyancy of an individual is determined by its body composition (i.e., the ratio of lipid to lean tissue; Biuw, McConnell, Bradshaw, Burton, & Fedak, 2003). Elephant seals frequently perform drift dives that are characterized by a prolonged phase of slow descent, during which seals drift passively through the water column, likely allowing the animal to rest and/or digest food (Crocker, Le Boeuf, & Costa, 1997). The rate of the vertical change in depth during these drifts dives (expressed in m/s) is largely affected by the buoyancy of the seal (a proxy of their body condition, Biuw et al., 2003). Fatter seals will sink less rapidly (or in certain cases become positively buoyant) than leaner seals, and so will exhibit drift rates that are less negative or in some cases positive. Success or failure PrCA rates can also be determined and linked to changes in swimming effort and changes in buoyancy and therefore in body condition (Abrahms et al., 2018; Richard, Cox, Picard, Vacquié‐Garcia, & Guinet, 2016).

While the dispersion of the first‐year juveniles southern elephant seals has already been described for two sub‐Antarctic islands (Field, Bradshaw, Burton, Sumner, & Hindell, 2005; McConnell, Fedak, Burton, Engelhard, & Reijnders, 2002; Tosh et al. 2015, 2012), and ontogeny in their diving behavior has only been described in studies at Macquarie Islands (Biuw et al., 2003; Hindell, Slip, Burton, & Bryden, 1992; Irvine, Hindell, Hoff, & Burton, 2000), none of these studies incorporated information taken from accelerometers. Moreover, juvenile dispersion and behavioral ontogeny from Kerguelen Island are unknown, and yet to be investigated.

The aim of this study was to describe the ontogeny of diving and foraging behavior in newly weaned southern elephant seals (*Mirounga leonina*) at Kerguelen Islands (Southern Ocean) during their first trip at sea following weaning. Using ARGOS satellite telemetry to relay summaries of accelerometer, dive and location data (Cox et al., 2017), we examined (Aim 1) the general spatial dispersion of juvenile southern elephant, (Aim 2) the development and adjustment of diving and foraging behavior in relation to changes in juvenile buoyancy, and in comparison with the behaviors of adult females from the same colony, (Aim 3) the physiological limits of juveniles, and if they were approached/exceeded in order to compensate for lower foraging efficiency and (Aim 4) how changes in buoyancy (i.e., body condition) influence the foraging decisions of juveniles, that is, to remain at sea or to return on land.

## 2 MATERIALS AND METHODS

### 2.1 Ethics statement and tag deployment

**Figure 1:**
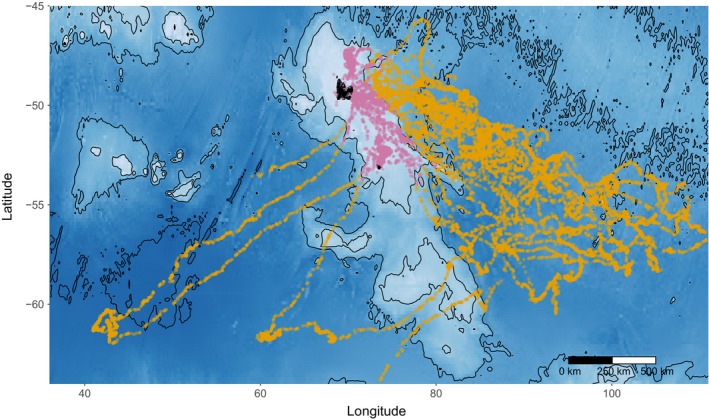
Post‐weaning foraging trips of juvenile southern elephant seals (*n* = 20) after departure from Kerguelen Island in 2014/2015. Colors indicate benthic and epi–benthic dives on the Kerguelen plateau (purple) or pelagic dives in the Southern Ocean (yellow). Dive locations are over‐layed onto a bathymetric map, where darker shades of blue indicate greater depths

All scientific procedures were approved by the Ethics Committee of the French Polar Institute. We equipped 20 juveniles (mean mass ± *SD* = 80 ± 18 kg; mean length ± *SD* = 139 ± 11 cm) with animal bio‐logging devices at Kerguelen Islands (49°204S, 70°204E, Figure 1) during November/December 2014 (Supporting information Table A1). Juveniles were fitted with a head‐mounted DSA tag (SCOUT‐DSA‐296, Wildlife Computers) as well as a SPOT tag (SPOT‐293 Wildlife Computers). This allowed the monitoring of juveniles beyond the lifespan of the DSA tags, as SPOT tags drain the battery at a slower rate. We also equipped nine post‐breeding adult females (mean mass ± *SD* = 267 ± 60 kg; mean length ± *SD* = 257 ± 38 cm) with time–depth recorders and accelerometers (TDR‐MK10‐X, Wildlife Computers) at the end of October between the years of 2010 and 2015. Post‐breeding females leave the colony only for 2 or 3 months in order to replenish their body reserve before coming back on land to molt, allowing for tag retrieval (Hindell & Perrin, 2009). Monitoring durations will thus be shorter for adult females compared to juveniles. Their diving and foraging behavior is used in this study as a reference in order to better understand how juvenile foraging and diving skills change in comparison with the general aptitudes of adult females. Adult males were not used because they are difficult to equip due to their large size (up to 3,700 kg whereas females only weight between 400 and 800 kg, Hindell & Perrin, 2009), and so no accelerometer datasets are available for male Kerguelen elephant seals. Moreover, they tend to display extreme diving skills that are much less comparable to juveniles than those of adult females (Hindell & Perrin, 2009). Following capture, all animals were anaesthetized using a 1:1 of tiletamine and zolazepam (Zoletil 100) injected intravenously.

### 2.2 Tag specifications and diving parameters

The DSA tags used for juveniles enabled a new method of collecting, abstracting, and transmitting accelerometer and dive data via the Argos satellite system (Collect Localisation Satellites—CLS, Toulouse, France). The advantages of this system are that real‐time diving data could be transmitted (c. 5 dives daily) negating the need for tag retrieval. Tags used on adult females were recovered when they returned to shore for their annual molt (January). The sampling rates of the juvenile tags were programed to record one dive every 2 hr, whereas for the adults, continuous dive records were obtained. During recording periods, for both juveniles and adults, pressure was recorded at a rate of 1 Hz and triaxial acceleration at 16 Hz. A full description of DSA tag specifications and on‐board processing algorithms can be found in Cox et al. (2017).

So that the two datasets would be comparable, high‐resolution datasets from the adults were processed using the same abstraction methods as implemented on‐board the DSA tags that the juveniles were equipped with (see Cox et al., 2017). Following this, all data were formatted to create a dive profile summary across three dive segments: descent, bottom, and ascent. In this study, the following dive metrics were analyzed: maximum dive depth (m), total dive duration (s), surface duration (s), descent and ascent swimming efforts (scaled by duration and used as an index of energy expenditure, in m/s^3^), and prey catch attempt rates during the bottom phase of a dive (PrCA; time spent catching prey in the bottom phase scaled by its corresponding duration). Total swimming efforts (before scaling by duration), reflected the sum of all accelerations (m/s^2^) on the lateral axis.

#### 2.2.1 Bathymetry—(Aim 1: Dispersion)

Bathymetry was obtained from the ETOPO1 bathymetric dataset, freely downloadable from NOAA at a one degree resolution (see Supporting information Appendix A1).

#### 2.2.2 Body Mass Index at departure—(Aim 2: Development)

We applied a linear regression between body mass at equipment/departure (see Supporting information Appendix A2 and Figure A1) and length at weaning, and took the residual values from that regression as the Body Mass Index (BMI, Supporting information Table A1). The calculated BMI was independent from body length and was used to compare individuals (Guinet, Roux, Bonnet, & Mison, 1998).

#### 2.2.3 Drift dives and drift rate (DR)—(Aim 2 & 4: Development & Buoyancy)

In this study, we consider changes in drifting vertical movement speeds (Drift Rates; DR) to be a proxy for changes in body composition (lipid vs. lean tissues) and therefore body condition (Biuw et al., 2003). Drift dive segments were identified using a filtering process similar to that described in Biuw et al. (2003) and Gordine, Fedak, and Boehme (2015), but modified to incorporate information from the accelerometer data on swimming effort and PrCA rates, as described below:

First, the first (descent) and last (ascent) segments of a dive were removed because seals typically start their drift at depth below 65 m after a first descent phase, and follow the drift segment with an active upward swimming phase. Second, all segments within which PrCA behavior was detected were removed. Third, according to the bimodal distribution in swimming efforts, segments with a swimming effort exceeding 4 m/s^3^ were removed. These two last filters are applied because the most likely purpose of a drift dive is to be inactive so as to rest and/or process food (Crocker et al., 1997). Fourth, the vertical speed of a segment was calculated by dividing the difference in depth by the time duration of the segment. Unrealistically, high speeds for a drift dive were identified by plotting the vertical speed distribution and comparing outliers to speeds reported in the literature. Vertical speeds below −0.3 m/s or above 0.6 m/s were thus removed. Dive segments with null vertical speed were also removed as there are thought to be linked to periods when an individual is resting on the ocean floor. Fifth, any drift segments that did not last at least 40% of the total dive time were removed, as a drift dive should last long enough for an individual to rest and/or process food. Drift rate (DR) are expressed in m/s, with positive values relating to positively buoyant periods, and negative values to negatively buoyant periods. In this study, we considered changes in DR (m/s) to be a proxy for changes in body composition (lipid vs. lean tissues) and therefore for changes in body condition (Biuw et al., 2003).

#### 2.2.4 Aerobic Dive Limit (ADL)—(Aim 3: Physiology)

The theoretical aerobic dive limits (tADL, Kooyman, 1989) of juveniles during their first 100 days at sea were estimated to be between 4 and 6 min (Hindell et al., 1999), and up to 18 min after 180 days at sea (Irvine et al., 2000). For adult females, tADLs are estimated to be between 28 and 30 min (Hindell et al., 1992). We used these threshold values to compare the dive durations of the juveniles in our study and quantify the theoretical proportion of anaerobic dives. To estimate the behavioral aerobic dive limits (bADL) and define a breaking point in the relationship between surface duration and dive duration, we followed the method of Kooyman (1989). Dives with durations exceeding this breaking point were considered as anaerobic.

### 2.3 Statistics

All data processing and analysis were performed in R, version 3.2.2 (R Core Development Team 2016). All numbers are given as mean ± *SD*, and the significance level was set at *p* = 0.05.

#### 2.3.1 Changes in diving behavior parameters: juveniles vs adults—Aim (2) & (3)

**Figure 2:**
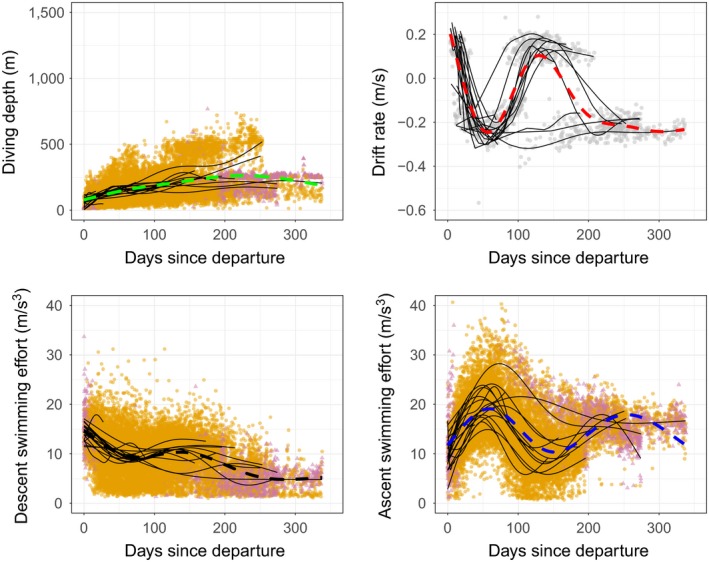
Changes in the diving behavior of juveniles (*n* = 20) through time. Dashed lines indicate estimates from the fixed components of GAMMs and thin black lines the individual random effects. The orange points indicate pelagic dives and triangular purple points represent benthic and epi–benthic dives

In order to study nonlinear responses of diving parameters relative to time since departure (Figure 2) or other diving parameters, we fitted generalized additive mixed‐effects models (GAMMs) via the *mgcv *package (Wood, 2017). Smooths were applied to the co‐variables (time since departure and diving parameters) in an interaction with age classes (adult vs. juvenile) to test differences. Smooths were fitted using cubic regressions splines with extra shrinkage to avoid over‐fitting. We included a corAR1 structure in all models to account for temporal autocorrelation (Zuur, Ieno, Walker, Saveliev, & Smith, 2009). A random effect of individual was included. To satisfy normality of residuals, response variables were transformed using Box–Cox power transformation when needed (Fox & Weisberg, 2011). GAMMs were fitted with a Gaussian error distribution and identity link function. Final model estimates were obtained using restricted maximum likelihood and then back‐transformed for plotting. All model outputs are in Supporting information (Tables A2–A5).

#### 2.3.2 Changes in dive duration and drift rate—Aim (2) & (3)

In order to estimate juvenile rates of change in dive duration and DR, we separated the monitoring period into 3 phases: departure, central, and return (Biuw et al., 2003). Rates of change were investigated during the first phase (departure) by fitting linear relationships (see Supporting information Figures A2 and A3). Individual coefficients (intercepts and slopes) for changes in dive duration or DR with time were retrieved using linear mixed‐effects models (LME) with a Gaussian error distribution via the *lme4 *package (Bates et al., 2017)**.** A random effect on the intercept and slope of individual was included (Zuur et al., 2009). For each LME, we used the MuMIn package (Barton, 2009) to estimate the marginal ($R_{m}^{2}$) and conditional ($R_{c}^{2}$) R squared values, which assess the fit of the fixed and entire model components, respectively. Slope and intercept values for each individual were then extracted, and the correlation between these values and co‐variables (such as the BMI) was tested by fitting a simple linear model (LM, Supporting information Figure A4 and A5).

## 3 RESULTS

### 3.1 Spatial Distribution—(Aim 1)

All juveniles left the natal colony in December 2014 (Supporting information Table A1). The majority headed south‐west of Kerguelen, except for two individuals, who headed south‐east (Figure 1). Within the first 100 days, both the DSA and SPOT tags stopped transmitting simultaneously for eight individuals, indicative of early mortality. The majority of dives were pelagic and occurred in oceanic waters, although some were benthic and occurred on the Kerguelen plateau (Supporting information Table A1). Three individuals (#140072, #140062 and #140066) became particularly faithful to the plateau by the end of the monitoring period, where they performed a large proportion of benthic dives (Supporting information Table A1). One individual #140070 also performed a high proportion of epi/benthic dives when leaving Kerguelen Island after weaning, however this individual, which did not reach oceanic waters and died only 20 days after departure.

### 3.2 Ontogeny of diving and foraging behavior: juveniles vs adults

#### 3.2.1 Changes in diving depth— (Aim 2)

**Figure 3:**
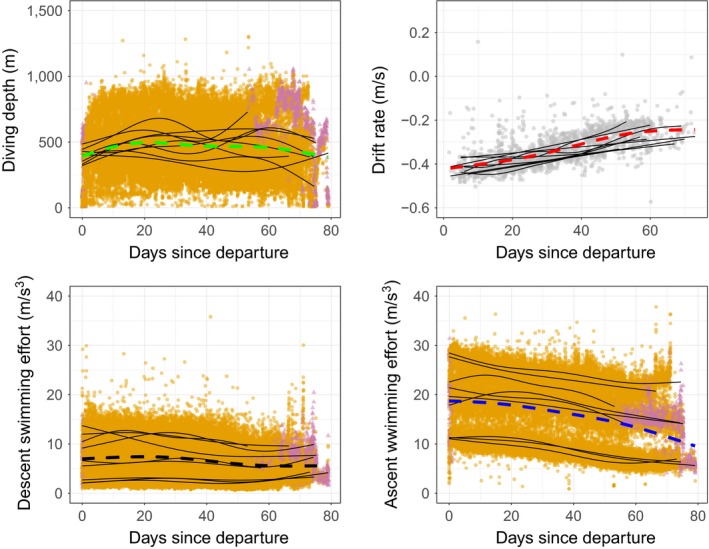
Changes in diving behavior in post‐molt adult female southern elephant seals (*n* = 9) through time. Dashed lines indicate estimates from the fixed component of GAMMs, and the thin black lines the individual random effects. The orange points indicate pelagic dives and triangular purple points represent benthic and epi–benthic dives

Juvenile diving depth (Figure 2) increased over time and reached an average of 115 ± 21 m at 50 days (max 456 m). Juveniles progressively increased their dive depths to a mean maximum of 227 ± 50 m after 8 months, whereas the mean diving depth for adult females was 474 ± 79 m across the entirety of their trip (Figure 3). The three individuals performing benthic dives on the Kerguelen plateau remained mostly in waters shallower than 250 m (max 768 m) (Figure 2).

#### 3.2.2 Changes in drift rate and swimming effort adjustment—(Aim 2)

**Figure 4:**
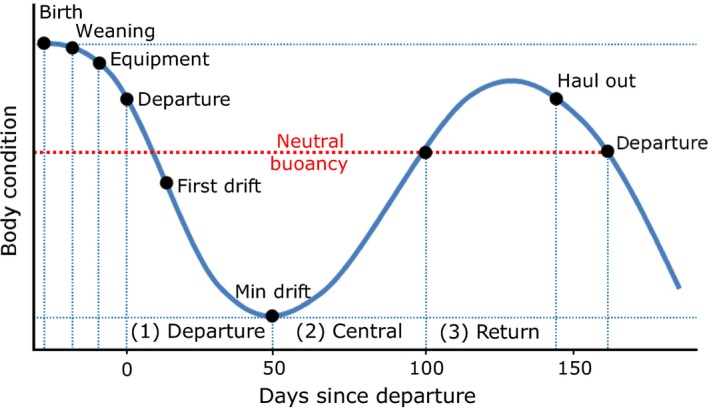
Schematic of typical changes in body condition of a juvenile during the first year at sea. Key stages, which are discussed in the results, are indicated by black dots. “Body condition” corresponds to drift rate values (m/s) estimated during drift dives and considered as a proxy of the body condition

In most cases, juvenile DR exhibited three successive phases (Figure 4): (1) the departure (up to 50 days) where they became negatively buoyant (2) the central phase (between 50 and 100 days) where they became positively buoyant, and (3) the return and haul out (after 100 days) where they became finally negatively buoyant (Figure 2).

DR became increasingly negative for all individuals during the first phase of the foraging trip. For nine individuals, the second phase corresponded to an increase in DR, culminating in a switch to positive buoyancy (DR > 0) after 93 ± 15 days, while the other 11 individuals remained negatively buoyant (DR < 0, Figure 2). The third phase corresponded to a second period of decrease in DR, although this observation is based on information from only three individuals (the tags of the six other individuals had stopped transmitting before this phase was reached). Adult females significantly reduced their ascent swimming effort related to gradual improvement of their body condition over time, without becoming positively buoyant (Figure 3). Juveniles reduced both their descent and ascent swimming efforts inversely related to changes in DR (Figure 2).

#### 3.2.3 Changes in foraging behavior— (Aim 2)

**Figure 5:**
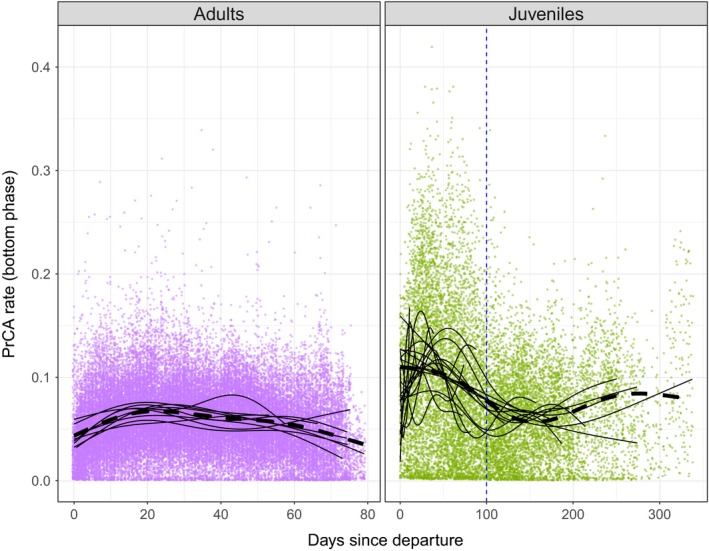
Comparison of changes in prey catch attempt (PrCA) over time for adult females (purple; left) and juveniles (green; right). Dashed lines indicate estimates from the fixed part of GAMMs and thin constant lines the individual random effects

During the first 100 days at sea following departure, juvenile PrCA rates were more than twice that of adults (Figure 5)**. **Juvenile PrCA rates decreased over the first 100 days, after which they stabilized at a level comparable to those observed for adults (Figure 5).

#### 3.2.4 Physiological diving limits in relation to swimming and foraging effort— Aim (3)

**Figure 6:**
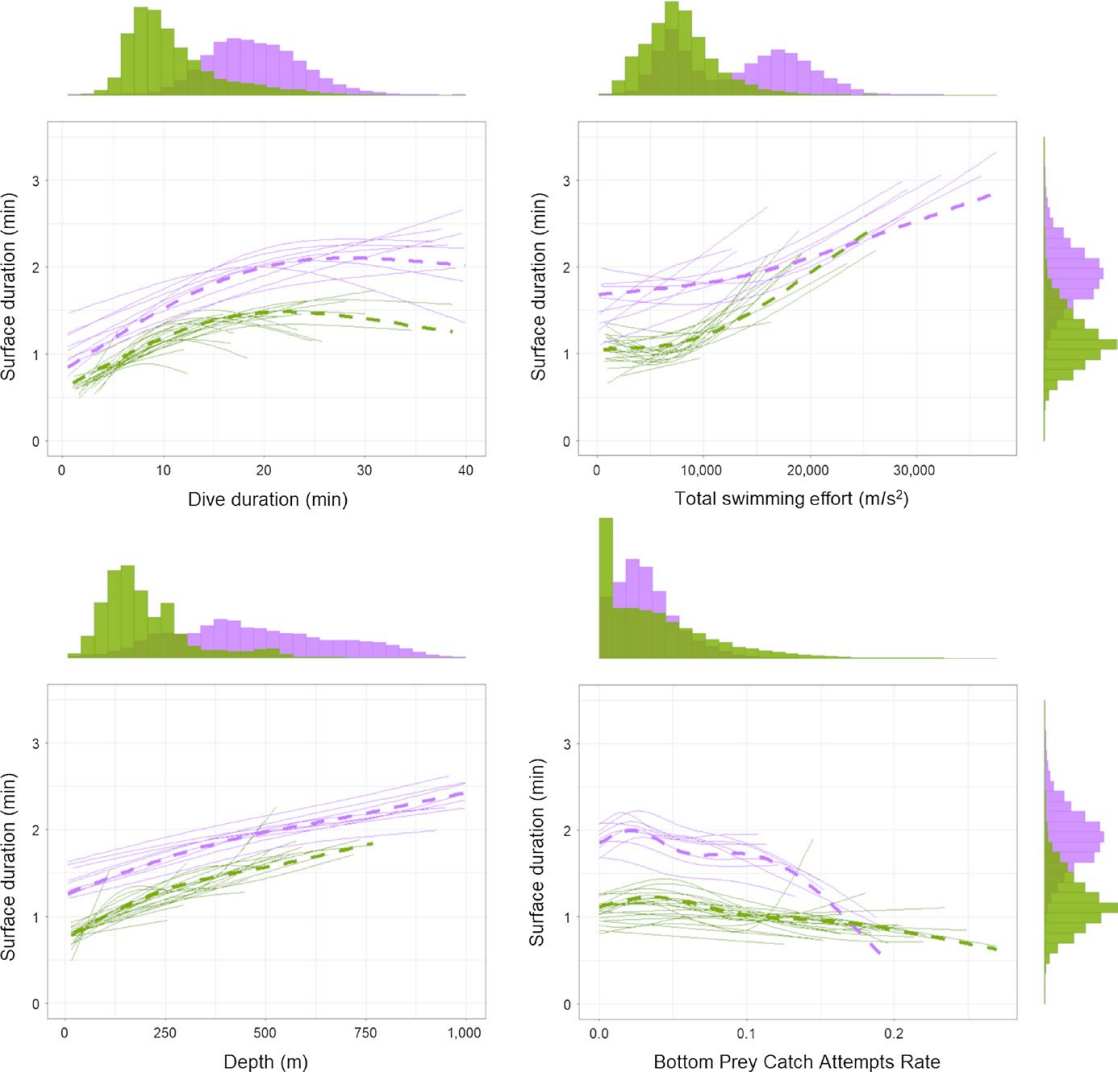
Comparisons of surface durations from adult females (purple) and juveniles (green). Dashed lines indicate estimates from the fixed part estimates from of GAMMs and thin constant lines the individual random effects. Extended surface durations (c. 1.3% of all dives; see Supporting information) were not included in the analysis

Surface durations were shorter for juveniles (1.15 ± 0.14 min) than adult females (2.0 ± 0.17 min, LM: *F* = 175.2, *p* < 0.001, *R* ^2^ = 0.86). There was a slight increase in surface duration as dive duration and depth increased (Figure 6). The shape of the relationship between the dive duration and surface duration did not reveal a behavioral ADL for juveniles, or for adults. Juveniles performed few (1.3%) extended surface durations (>3.5 min, see Supporting information Appendix A3). The proportion of dives exceeding the theoretical ADL changed according to trip phase (Figure 4): (1) 76% ± 22% of departure phase dives exceeded 6 min; (2) 16% ± 10% of central phase dives were above 10 min; and (3) 20% ± 5% of return phase dives were above 18 min. Average dive durations for juveniles were 12.9 ± 5.0 min, maximum 34.7 min. In contrast, adult female dive durations averaged 18.5 ± 5 min. For a given dive duration, juveniles exhibited shorter post‐dive surface durations compared to adult females (Figure 6). Post‐dive surface durations increased with increasing swimming effort performed during a dive. The slope of this relationship was steeper for juveniles (Figure 6). Surface durations decreased with increasing PrCA rates during the previous dive for both adult females and juveniles (Figure 6).

### 3.3 Juveniles foraging trip phases in relation to changes in body condition—(Aim 4)

#### 3.3.1 On land before departure

Between tag deployment and departure to sea, juveniles spent on average 9.7 ± 6.6 days on land (Table A1). The time spent on land between equipment and departure (Supporting information Table A1) was positively correlated with BMI at equipment (LM: *F* = 13.22, *p* < 0.02, *R* ^2^ = 0.39, Figure A6).

#### 3.3.2 Departure

Initial DR values after departure were positive for 17 juveniles (0.15 ± 0.04 m/s) and negative for 3 (−0.21 ± 0.02 m/s) and were unrelated to BMI at departure (LM: *F* = 0.19, *p* = 0.67, *R* ^2^ = 0.01). The decrease in DR over time (individual slopes), estimated from random effects (LME: $R_{m}^{2}$ = 0.47; $R_{c}^{2}$ = 0.81), was negatively correlated with initial observed DR values per individuals (fatter individuals were loosing their condition faster, *F* = 41.9 *p* < 0.01, *R* ^2^ = 0.69, Supporting information Figure A4). Dive durations of juveniles during the departure phase were positively correlated to departure lengths (LM: *F* = 17.1, *p* < 0.01, *R* ^2^ = 0.46, Supporting information Figure A5), but not with departure BMI (LM: *F* = 2.2, *p* = 0.158, *R* ^2^ = 0.06). Increases in dive durations during the first 50 days, estimated from random effects (LME: $R_{m}^{2}$ = 0.05, $R_{c}^{2}$ = 0.53, Supporting information Figure A3), were positively correlated to BMI at departure (LM: *F* = 6.5, *p* = 0.02, *R* ^2^ = 0.23, Supporting information Figure A5).

#### 3.3.3 Return

**Figure 7:**
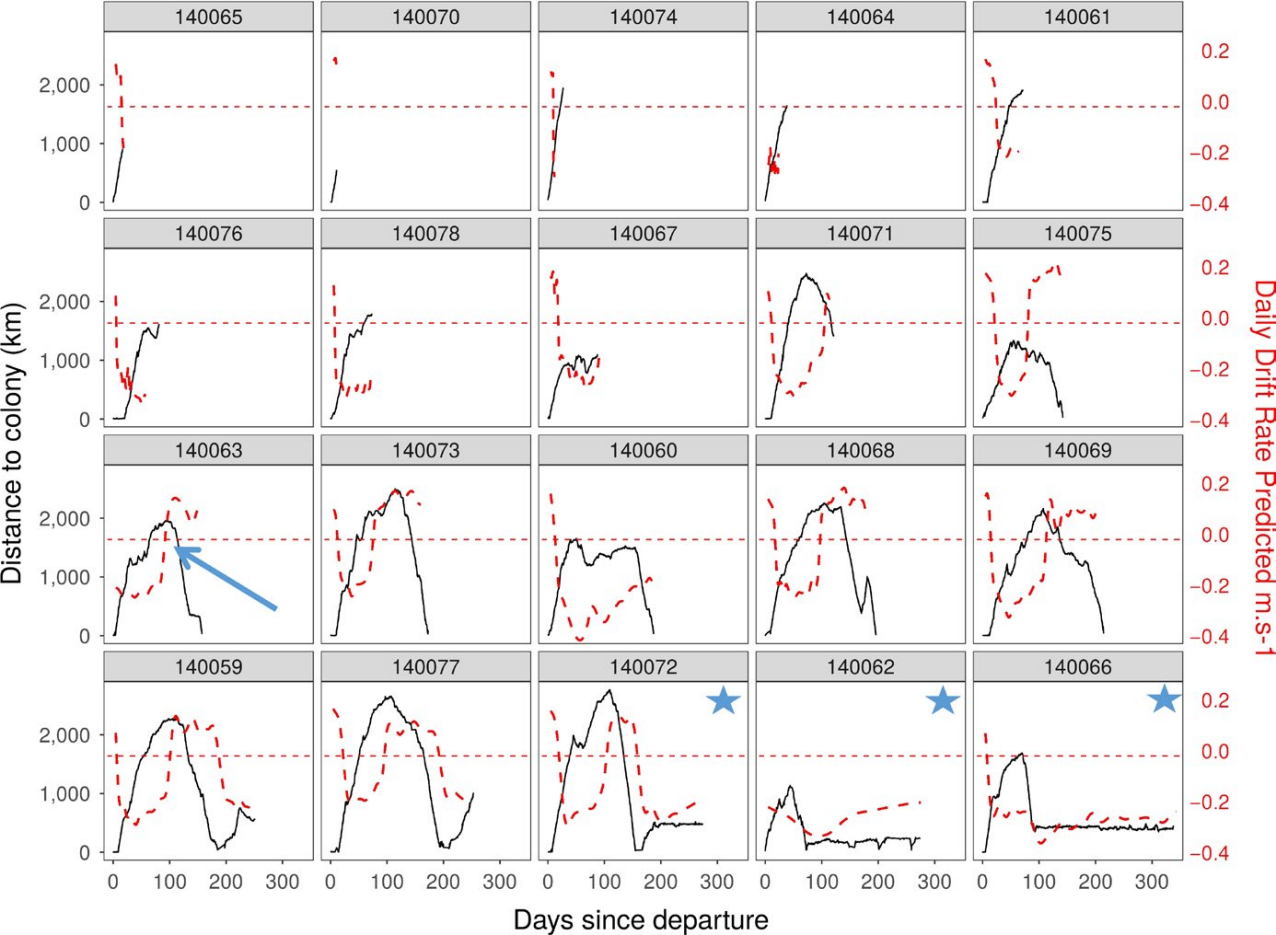
Relationships between distance to the colony (solid black lines) and body condition (dashed red lines) according to time for each juvenile. The horizontal red dashed line at 0 m/s indicates neutral buoyancy. Figures marked with stars indicate individuals that foraged on the Kerguelen plateau at the end of their trip. The blue arrow indicates an example of when an individual started the return phase of its first trip at sea following a switch to positive buoyancy

The transition to positive buoyancy was concomitant with the return phase for eight of the nine individuals who became positively buoyant (Figure 7 and Supporting information Figure A7, #140075, 63, 73, 60, 68, 69, 59, 77, and 72), all of which were foraging in oceanic waters. Turning points, that is when animals started to swim back to shore, ranged between 500 and 2,600 km away from the departure location. DR tended to decrease in the return phase (Figure 7).

#### 3.3.4 Haul outs and habitat changes

The individuals who spent time foraging on the Kerguelen plateau (2 males #140072, 62 and 1 female #140066) were negatively buoyant while doing so, while all individuals managed to become positively buoyant were foraging in oceanic waters (Figure 7). Haul out duration was shorter for individuals on the plateau (2.9 ± 2.59 days vs. 8.7 ± 4.4 days, Kruskal–Wallis: *X* ^2^ = 11.6, *p* < 0.01, see Supporting information Appendix A4 and Table A6). Comparing PrCA rates performed in oceanic vs plateau waters revealed no significant differences (LME, *F* = 1.93, *p* = 0.164, $R_{c}^{2}$ = 0.00, $R_{m}^{2}$ = 0.18).

## 4 DISCUSSION

Innovative on‐board satellite data relay tags (Cox et al., 2017) permitted us to retrieve dive data (depth, duration, and surface durations) alongside accelerometer data (swimming effort and PrCA) from a free‐ranging marine predator in real time. Despite our relatively small sample size (20 individuals) that diminished with time (probably due to early mortality; nine individuals stopped transmitting within 100 days) we obtained one of the longest behavioral monitoring periods among deep‐diving species. These data allowed us to describe changes in juvenile southern elephant seal diving behavior, foraging efficiency, body condition, physiological limit, and foraging habitat during their first year at sea following weaning. This study highlights the importance of using accelerometer data (e.g., swimming effort and PrCA) in order to understand the mechanisms driving ontogenetic changes in the foraging behaviors of deep‐diving marine top predators.

### 4.1 Differences between juvenile and adult female diving behavior

#### 4.1.1 Distribution, dive depth, and dive duration

After departure from Kerguelen Islands, juvenile dispersion patterns were similar to those previously described for Macquarie Island (McConnell et al., 2002) and were generally directed toward the south‐east (Figure 1). Juveniles performed shallower and shorter dives compared to adult females from either post‐breeding (our study) or post‐molt trips (Bailleul et al., 2007).

#### 4.1.2 Buoyancy and swimming effort

Female post‐breeding elephant seals are known to make adjustments in swimming effort according to changes in buoyancy (Richard et al.., 2014). Transportation costs during transit (descent and ascent phases) are minimal at neutral buoyancy, but a 1% deviation from this can induce a 20% increase in swimming effort across a dive, with direct consequences on dive duration (Richard et al., 2014). Remarkably, juveniles were also able to finely adjust their swimming efforts in response to changes in buoyancy (Figure 2). To our knowledge, this is the first time that this adjustment has been reported for a juvenile diving predator. This suggests that juvenile southern elephant seals are rapidly able to optimize behavior (swimming effort or dive duration) during the transit phases of a dive to benefit from buoyant forces. This behavioral change implies the possible presence of rapid learning, adapted behavioral posture, and instinctive adjustment against drag forces (Williams et al., 2000).

#### 4.1.3 Change in foraging success

Immediately following departure from Kerguelen Islands, juveniles exhibited higher PrCA rates compared to adult females (Figure 5). This was accompanied by a decrease in buoyancy (Figure 2), suggesting a comparatively low success rate. This could be the result of (a) poor prey handling skills, (b) opportunistic behavior, and/or (c) reliance on smaller and less nutritious prey (Marchetti & Price, 1989). There is support for the latter of these hypotheses (c) as a number of studies (Lübcker et al., 2017; Walters et al., 2014) have shown that the diet of juvenile elephant seals during their first year at sea is predominantly composed of krill (*Euphausia sp.*).

After 100 days at sea, juvenile PrCA rates decreased significantly to levels equivalent to those observed for adults. However, despite these decreased PrCA rates, most juveniles became positively buoyant suggesting a shift in diet and/or an improvement of foraging skill. At this time, which corresponds to the end of the austral summer, nycthemeral behavior of juveniles became much more pronounced (see Supporting information Appendix A5 and Supporting information Figure A8). This progressive change toward increased nycthemeral behavior is associated with increased diving depth. This suggests that with an improvement in dive abilities, juveniles are becoming more and more able to follow prey resources as they deepen during daylight hours. This change is also correlated with an improvement in body condition and a reduction in PrCA rate (Figure 5). However, even after almost one year spent at sea, the juveniles never reach diving aptitudes equivalent to that of adult females (depth, Figure 2, dive duration, Figure 6). On average, after 8 months at sea, the diving aptitude of juveniles was about 50% of the diving depth of adult females and 70% of dive duration. As such, juveniles are likely not able to access the same prey densities and/or species as adults.

#### 4.1.4 Physiological limits

Because of their smaller size, juveniles should have a higher metabolic rate than adults (Boyd & Hoelzel, 2002), and face physiological limitations that constrain their diving ability, such as oxygen storage capacity in blood and muscle. We noted that juveniles exceeded the tADL for many of their dives, while no bADL could be detected (Figure 6). This suggests an under‐estimation of their physiological limits, as appears also the case for other species (e.g., in Northern elephant seals, Hassrick et al., 2010).

Absolute surface durations relative to a given dive duration were shorter in juveniles compared to adults, which has been observed in other studies of southern elephant seals (Hindell et al., 1992; Irvine et al., 2000). In contrast, juveniles of several other air‐breathing marine top predators perform longer or equal surface durations compared to adults, probably because they present lower diving skills, for example, juvenile king penguins (Orgeret et al., 2016), Weddell seals (Burns, 1999) and Australian sea lions (Fowler, Costa, Arnould, Gales, & Kuhn, 2006). This could indicate that because of their smaller size, juvenile elephant seals were able to replace their oxygen debt more quickly than adults (Butler, 2004). For both juveniles and adults, surface durations increased with the total swimming effort performed during the previous dive. This was especially true for juveniles, where the slope of the relationship between the absolute swimming effort of a dive and the following surface duration was steeper than that of adults (Figure 6). Recent studies suggest that there is a positive relationship between surface duration relative to total dive duration and swimming effort during the previous dive in adult female elephant seals (Day, Joumaa, Bonnel, & Guinet, 2017; Génin et al., 2015; Jouma'a, 2016). This suggests that slight increases in swimming effort cause juveniles to increase their time at the surface in order to breathe and recover, even if they need to spend less time at the surface compared to adults. Day et al. (2017) showed that PrCA rates positively influenced post‐dive surface duration in adult females, likely because catching prey induces higher energy expenditure through increased swimming effort, turning movements, and digestive costs. The adult females we studied showed the contrary and tended to decrease their recovery time at the surface after a high level of PrCA rates (> 0.1, Figure 6) likely to minimize non‐foraging time. This was also the case for juveniles, even if the slope coefficient of the relationship was lower compared to adult females. Many diving predators are known to increase their dive descent angles when they have found food during the previous dive (e.g., king penguins, Hanuise, Bost, & Handrich, 2013), and this is accompanied by an increase in foraging effort and a reduction in surface duration in king penguins, but increased post‐dive surface duration in fur seals (Viviant, Monestiez, & Guinet, 2014). Because of their higher energy requirements, juveniles might be less capable of storing oxygen reserves than adults. It seems therefore, that juveniles are not able to decrease as well their surface duration following highly successful foraging dives in order to return to foraging depths more rapidly.

### 4.2 Changes in buoyancy in relation to foraging trip and habitat

#### 4.2.1 Time to departure and body condition

Once a juvenile is weaned and its mother has departed to sea, it will spend 4–6 weeks fasting on land and/or within inshore waters immediately surrounding its natal colony. This period is critical to ensure a transition to pelagic life (Castellini, 1996). Juveniles with a higher BMI were found to leave the colony later than lighter ones, which is consistent with the findings of Arnbom, Fedak, Boyd, and McConnell (1993). No relationship was found between BMI and buoyancy at departure, which suggests that BMI does not provide precise information on the real body composition of juveniles, which to be assessed (percentage body fat, lean, and bones tissues) prior to departure would require isotopic dilution techniques (tritiated water, deuterium). However, juveniles appear to leave on foraging trips only when fat reserves are sufficient for adequate thermoregulation and sufficient body‐stores (Guinet, 1992), to insure a few weeks of survival, but not so great as to veer too far away from neutral buoyancy would increase the cost of transport (Williams et al., 2000).

#### 4.2.2 The foraging trip: three important phases

As described in previous studies (Biuw et al., 2003; McConnell et al., 2002), changes in body condition after departure allowed the delineation of three important phases in a juvenile's first trip to sea, namely: (1) departure, (2) central phase, and (3) return (Figure 4).

##### Phase 1: Deterioration of body condition

Dive duration over time increased more rapidly with increasing BMI at departure (Supporting information Figure A5). This is consistent with previous findings (Hindell et al., 1999; Irvine et al., 2000), including a demographic study by McMahon et al. (2015) which indicated that weaning mass and condition are important factors in the survival of first‐year juvenile southern elephant seals. Individuals with a high BMI may have more time to develop their physiological aptitude compared to those with a lower BMI. The first phase of their offshore foraging trip was associated with a decrease in body condition for all juveniles. Juveniles of higher buoyancy at departure experienced a more rapid deterioration in body condition than other individuals (Supporting information Figure A4). This could be explained by the fact that more buoyant juveniles encounter higher energetic costs during diving compared to individuals close to neutral buoyancy. Following this first period of body condition decline, the tags of eight juveniles stopped transmitting. This was within the first 100 days spent at sea, and all but one of them (dying within two weeks of departure) were negatively buoyant when they died. This high rate of early mortality suggests that the beginning of a juveniles foraging life is particularly challenging. This change in buoyancy is thus a constraining phase and probably explains the high mortality that occurs in the first year of a southern elephant seals' life (~40%, Pistorius, Bruyn, & Bester, 2011).

##### Phase 2: Improvement of body condition

After the first phase, among the 12 individuals surviving more than 100 days, nine were foraging successfully enough to become positively buoyant, while three remained negatively buoyant. As shown in Figure 7, this transition may act as a signal for return to land. Remaining close to neutral buoyancy is optimal for minimizing transport costs. However, when individuals are near neutral buoyancy, small changes in density lead to large changes in buoyancy (Biuw et al., 2003). Increasing transportation costs related to increased buoyancy could trigger the decision to come back ashore to fast. As individuals get older and grow in size, exceeding neutral buoyancy appears to be less frequent. Seals therefore remain at sea for longer durations, allowing them to forage further from land and to explore and discover new important foraging areas (Field et al., 2005). Juveniles returning ashore positively buoyant were found to remain on land for a longer duration compared to those arriving negatively buoyant. This further supports the idea that buoyancy acts as a signal to return to shore to fast and may control to some extent the duration of the visit ashore. Adjusting buoyancy could be one of the functions of these haul outs (Field et al., 2005).

##### Phase 3: A change in foraging habitat

After their first trip at sea, three individuals (#140066, #140062 and #140077) remained on the Kerguelen plateau. Two of them did not manage to become positively buoyant during this time. Despite our small sample, foraging success experienced early in life appears to be influenced by a choice of two strategies: benthic vs pelagic. The diving behavior and foraging site fidelity of immature southern elephant seals (<4 years old) are poorly known. Describing these would be important to the understanding of the proximate causes of ontogenetic shifts in habitat preference occurring early in life (Chaigne, Authier, Richard, Cherel, & Guinet, 2013).

While juveniles that foraged in oceanic waters became positively buoyant, those that foraged benthically on the Kerguelen Plateau remained negatively buoyant (Figure 7). Several nonexclusive hypotheses could explain such differences in foraging efficiency, that is, the ratio between energy intake and expenditure, a greater foraging cost, diet composition, and prey size, and energy content.

## 5 CONCLUSION

This study highlights the importance of using accelerometer data relayed by satellite to relate foraging skills and changes in body condition to changes in foraging decisions and habitats (plateau vs. ocean). This could partially explain ontogenetic processes in habitat shift, alongside spatial segregation in marine diving predators. Because of their smaller size, juveniles were restricted in their foraging aptitude compared to adults. However, they improved their diving and foraging efficiency and showed a remarkable ability to adjust their diving behavior according to their change in buoyancy. Future studies are needed, to increase the sample size to be able to make population inferences, and to ascertain the value of different habitats and the importance of physiological development alongside the impacts these have on the mortality rates of juveniles. This understanding could be critical in predicting changes in population dynamics of air‐breathing deep‐diving predators.

## CONFLICT OF INTEREST

None Declared.

## AUTHORS CONTRIBUTION

C.G.: study conception and design; F.O., S.L.C., H.W., and C.G.: analysis, interpretation, drafts, and revision. All authors approved the final version of the manuscript and agree to be held accountable for the content therein.

## DATA ACCESSIBILITY

All diving data are available in the Dryad public data at https://datadryad.org/ https://doi.org/10.5061/dryad.qq6mb07
