# Supplementary material for: Body condition influences ontogeny of foraging behavior in juvenile southern elephant seals
Source: Ecol Evol. 2018 Dec 18;9(1):223–36. doi: 10.1002/ece3.4717 (PMC6341977; doi:10.1002/ece3.4717)
Supplement: Supplementary file 1 [file ECE3-9-223-s001.docx]

**Body condition influences ontogeny of foraging behaviour**

**in juvenile southern elephant seals**

**Florian Orgeret, Sam Cox, Henri Weimerskirch and Christophe Guinet**

**Appendix**

**A1. Bathymetry**

Bathymetry data were obtained from the ETOPO1 bathymetric dataset, freely downloadable from NOAA at a one degree resolution. For each dive, the spatially matched bathymetry was extracted. Dives registered at or deeper than their spatially matched bathymetry were considered benthic dives, whilst dives with maximum depths that occurred above 90% of the bathymetry were considered as epi-benthic dives (Table A1 and Figure 1). Dives performed within the 2000 m depth contour line were considered to be on the Kerguelen plateau (O’Toole, Hindell, Charrassin, & Guinet, 2014) but were not necessarily benthic or epi-benthic (Figure 1).

**A2. Body Mass Index at departure**

Between 2011 and 2016, 630 juveniles were measured and weighed at weaning at Kerguelen Island. Assuming that their length would not change between weaning and departure to sea, and using a mean fasting duration of 6 weeks (42 days) prior to departure (McConnell, Fedak, Burton, Engelhard, & Reijnders, 2002) we calculated a predicted mass at departure following (Guinet, 1992):

M_d_ = M_w_ – (0.0048 * M_w_ + 0.3031) * 42 (Eq. 1)

where M_d_ is the mass at departure (to be estimated) and M_w_ is the mass at weaning. We then applied a linear regression to body mass at departure and length at weaning and took the residual values from that regression as the Body Mass Index (BMI, Fig. A1). The relationship between BMI and length could then be considered as independent and thus comparable between individuals (Guinet, Roux, Bonnet, & Mison, 1998, Fig. A1). The departure dates of individuals equipped with a tag were taken as the day when they did not return to land and began to perform dives deeper than 50 m. We then calculated mass and BMI at departure following the methods as mentioned above (Table A1).

**A3. Extended Surface Intervals**

Juveniles performed extended surface intervals (ESI > 3.5 min) in 1.3% (n=211) of all dives. The mean duration of these surface intervals was 8.1 ± 2.5 min with a maximum of 31.9 min. We did not find any relationship between extended surface duration and other diving parameters such as depth, dive duration, PrCA rates, swimming effort or time since departure. However, the frequency of extended surface intervals per individual increased slightly at night (6.8 ± 4.6 ESI at night vs 2.9 ± 2.2 ESI during the day, LME: z = 4.7 p < 0.01).

Other studies have described similar durations of extended surface intervals (e.g. (Mark A Hindell et al., 1999; Irvine, Hindell, van den Hoff, & Burton, 2000; Le Boeuf, Morris, Blackwell, Crocker, & Costa, 1996), even in adults (Hindell, Slip, & Burton, 1991). These surfaces intervals may be related to the maintenance of some organs that turn off during diving (e.g. kidney or liver, see Hindell et al., 1991).

**A4. Haul outs**

Over the entire transmission period of the SPOT tags, juveniles performed several trips to sea. Individuals made several mid-winter haul outs (Table A6), usually between June and August when they fasted on land for few days before returning to sea. The individuals who made foraging trips to the Kerguelen plateau hauled out more often than those that remained offshore in oceanic environments (Kruskall-Wallis, KW, X² = 3.9, p = 0.049, 2.9 ± 3.1 haul outs for neritic individuals versus 1.1 ± 0.3 for oceanic). One individual (#140066) performed as many as 10 haul outs, most of them on Heard Island, which was very close to its foraging grounds at the southern part of the Kerguelen plateau. Individuals who foraged on the Kerguelen plateau remained negatively buoyant (Figure 7), and the opposite was true of individuals performing oceanic trips. Haul out periods were also shorter for neritic individuals than oceanic individuals (2.9 ± 2.59 days *vs* 8.7 ± 4.4 days, KW: X² = 11.6, p < 0.01).

**A5. Nycthemeral Dives**

We used the *maptools* R package, (Bivand et al., 2017) to associate each dive location to its associated solar azimuth and time of day (day or night) according to the nautical twilight definition (i.e. when the sun is geometrically 12 degrees below the horizon). A mean depth was calculated for each day since departure and used to assess differences in behaviour between night and day (nycthemeral behaviour).

As soon as they left their natal colony, juvenile seals exhibited a nycthemeral diving pattern, performing deeper dives during the day. The differences between daytime and nighttime diving depths increased over time, most markedly after 100 days at sea (i.e. ~2 months after departure which corresponds to the beginning of the Austral winter). This nycthemeral behaviour was less obvious, but still present for juveniles foraging on the Kerguelen plateau (Fig. A8).

Seasonal effect on nycthemeral behaviour

Juvenile seals started exhibiting a nycthemeral diving pattern soon after leaving their natal colony, with differences between day and night diving depths increasing over time, most markedly after c. 100 days i.e. the beginning of the austral winter (Fig. A8).

**Figures**


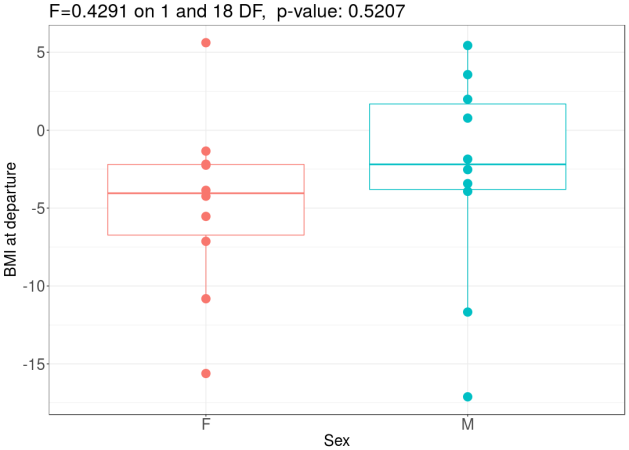

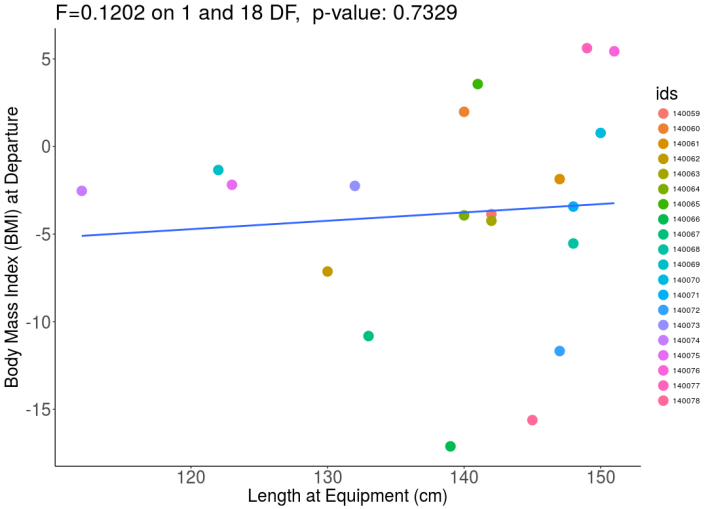

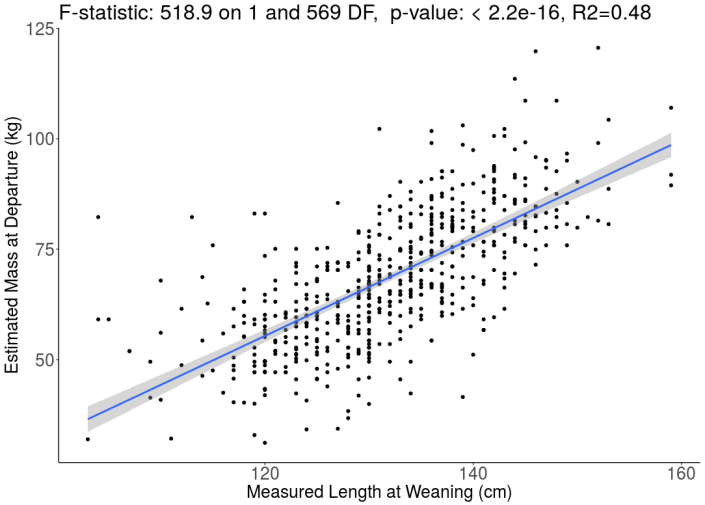


**Figure A1:** Top left is the linear regression allowing extraction of residuals (Body Mass Index, BMI) for each individual. The top right figure shows the relationship between the BMI and length at departure, the relationship was not significant. The boxplot of BMI, on the lower lower left, shows no significant difference between juvenile females and juvenile males.


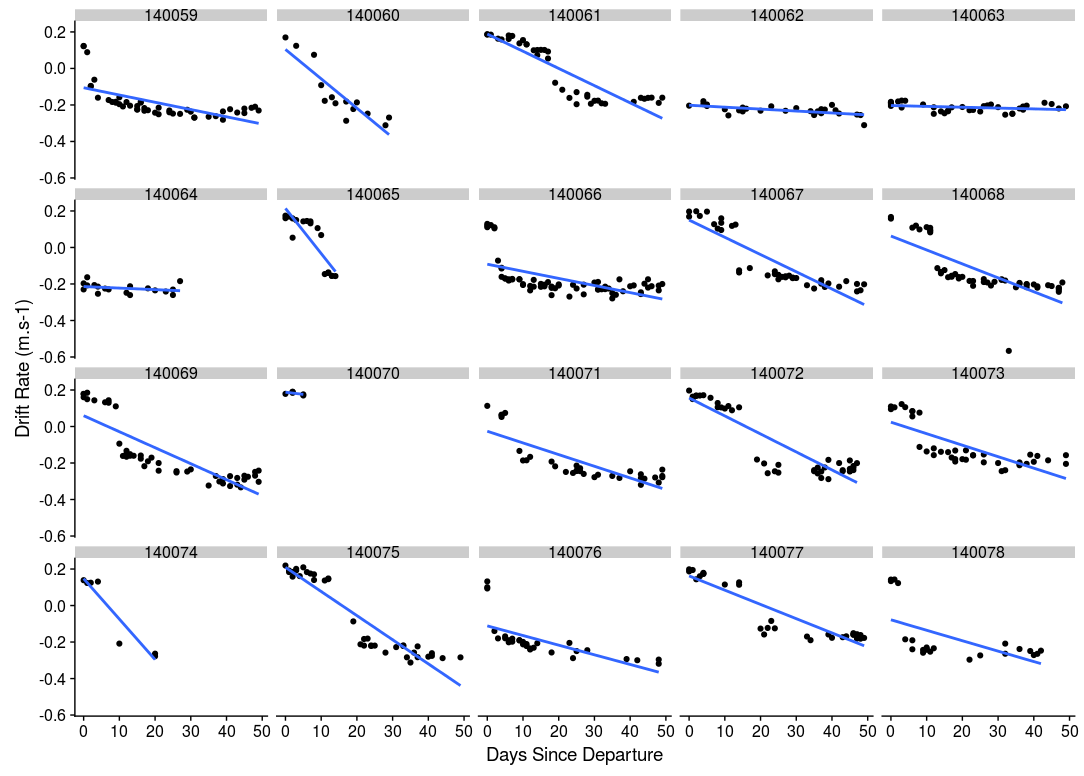


**Figure A2:** Random slope/intercepts linear mixed models outputs for the changes in drift rates during the first 50 days at sea for each juvenile. Black points are the raw data. See Figure A7 for the complete time series.


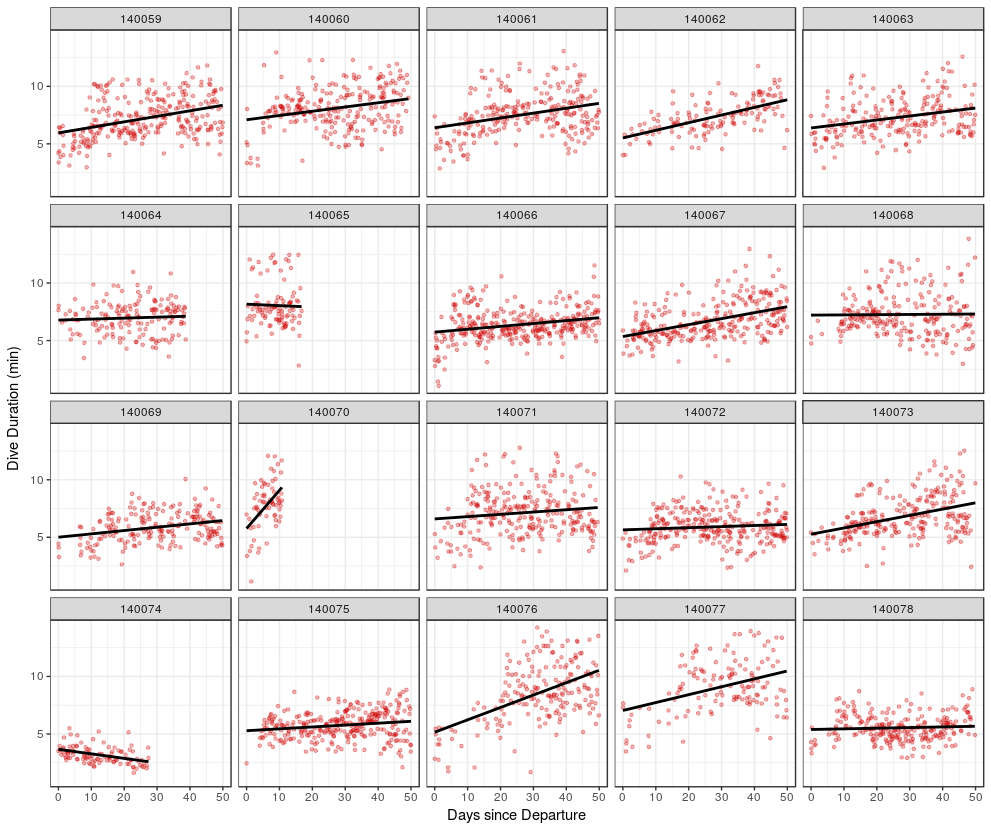


**Figure A3:** Random slopes/intercepts linear mixed models outputs for the changes in dive durations during the first 50 days at sea for each juvenile. Red dots are the raw data.


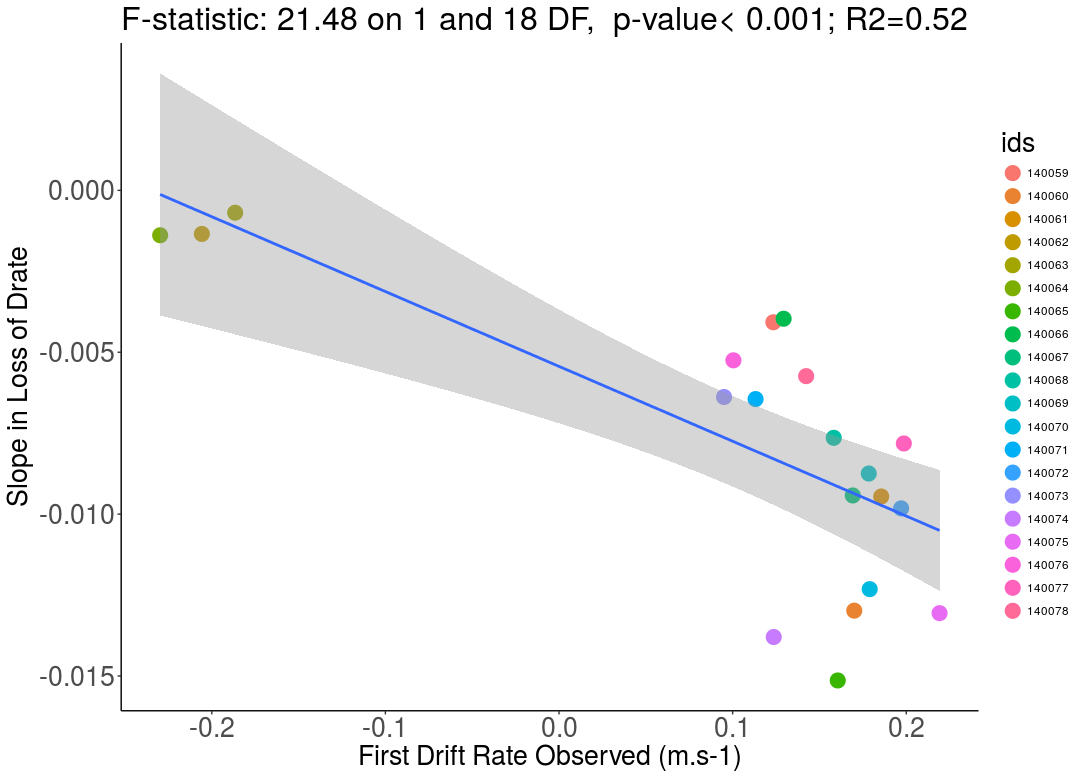

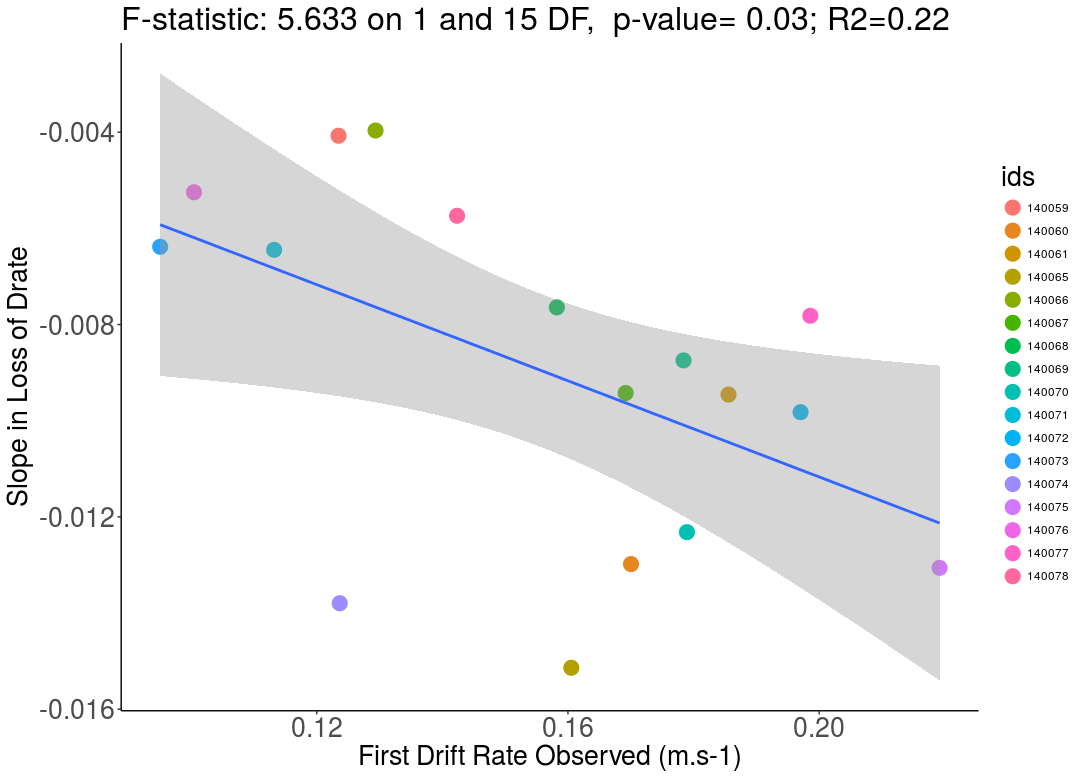

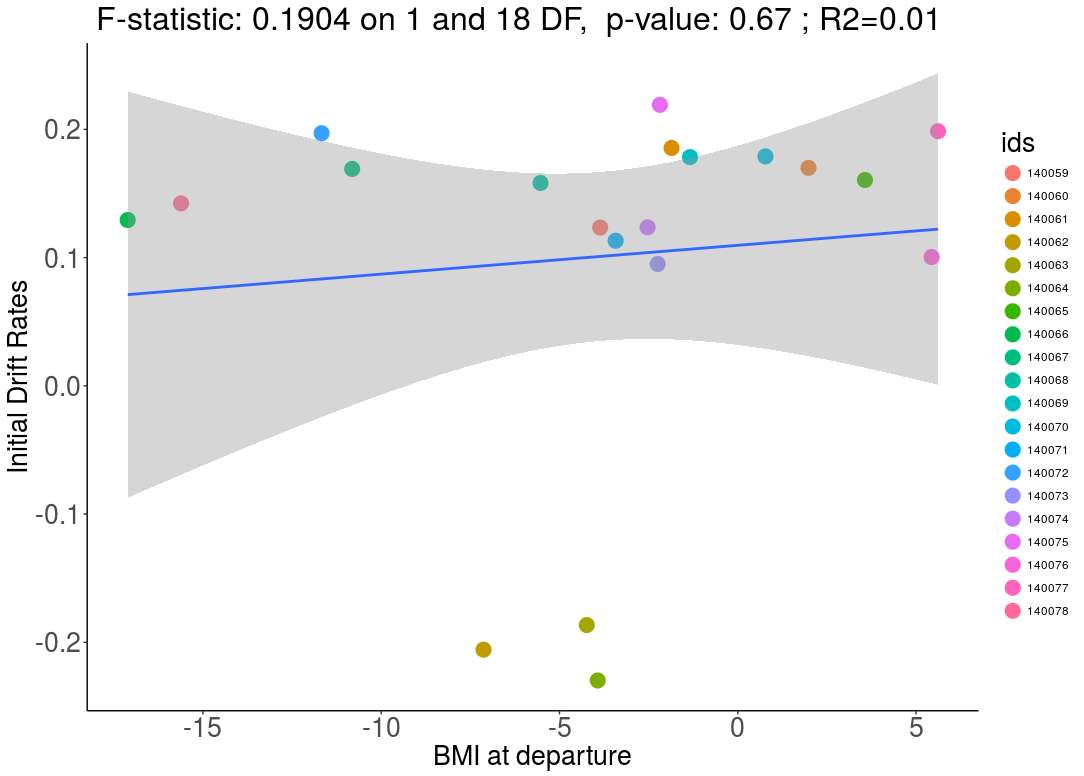


**Figure A4:** Initial drift rates BMI do not have a strong correlation (top left), however the slope of the changes in drift rate during the first 50 days correlates to the inital drift rate values observed per individual (top right). This correlation is stronger if the three outliers (#140062, #140063 and #140064) with negative the initial drift rates are removed (lower left). No differences were found between males and females when comparing drift rates slopes (F=1.9, p=0.189) and initial observed drift rate values (F=0.1422, p=0.710)


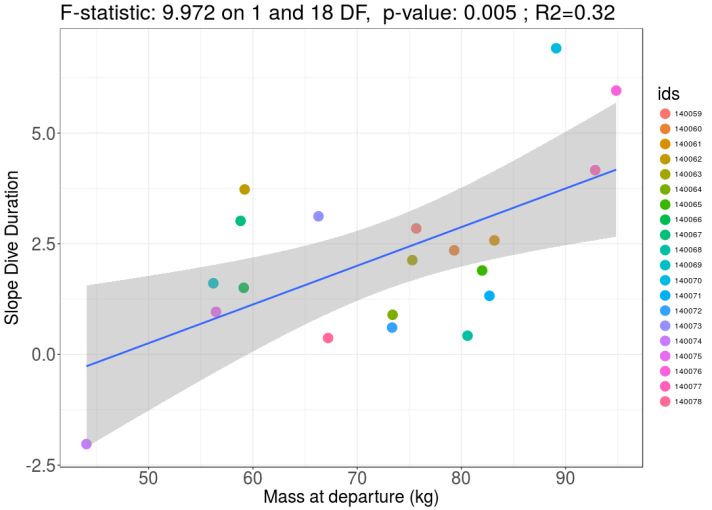

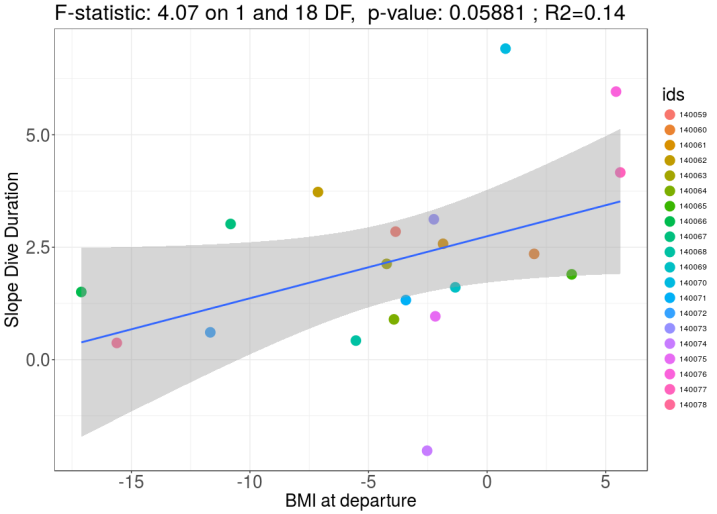

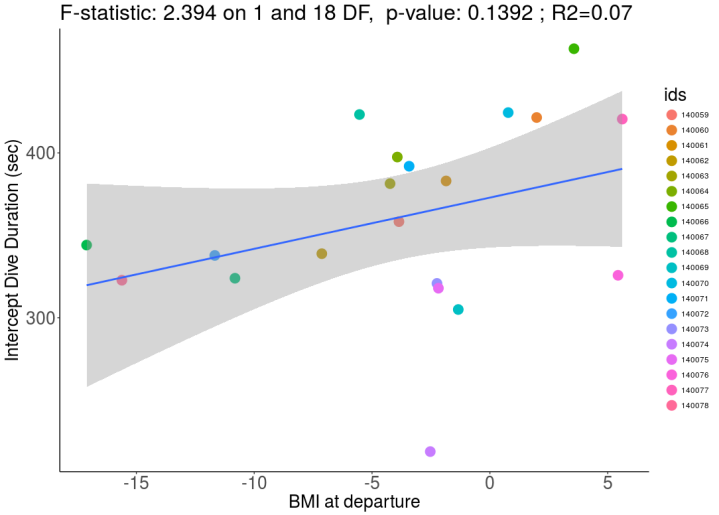

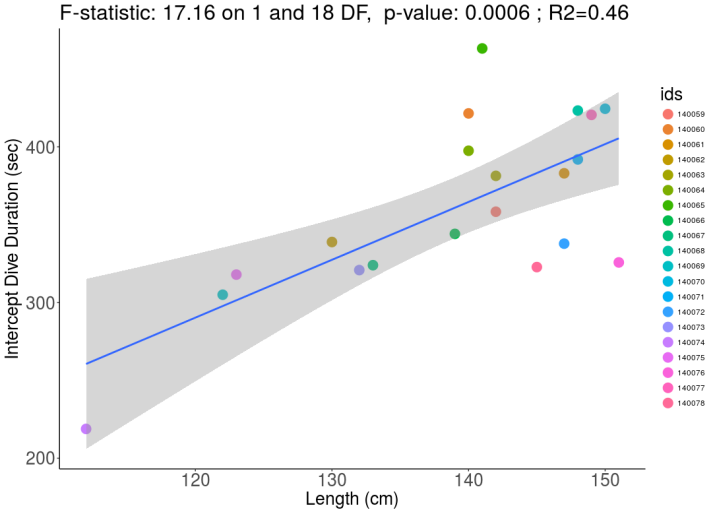


**Figure A5:** The duration of the first dive observed per juvenile is correlated to the body length (top left) but not to their BMI (top right). The changes in dive duration during the first 50 days is not correlated to the BMI (lower left) but is correlated to the body mass estimated at departure (lower right).


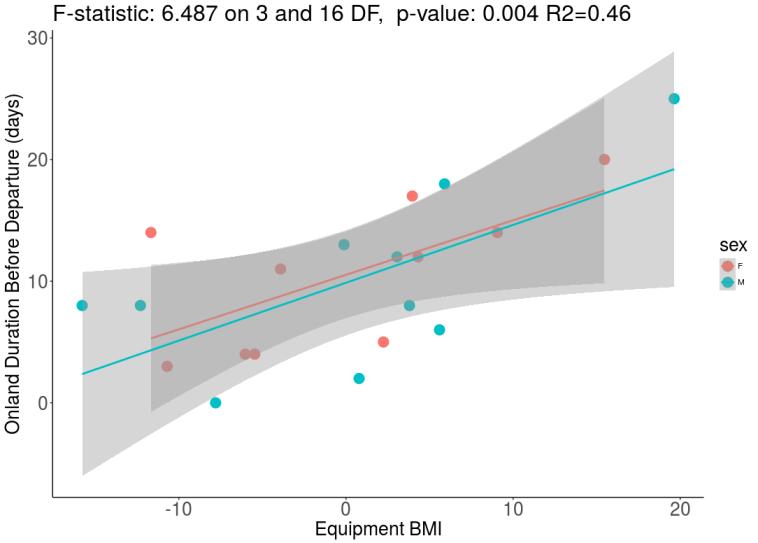

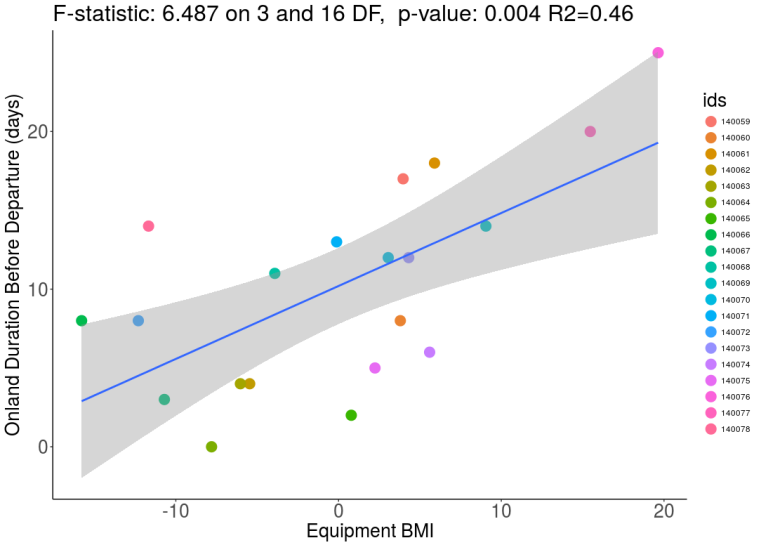


**Figure A6:** The relationship between time spent on land before departure and BMI estimated at equipment is significant (right) but there is no difference between male and female juveniles (left, intercept: t = -0.196, p = 0.847; slope: t = -0.042, p = 0.967).


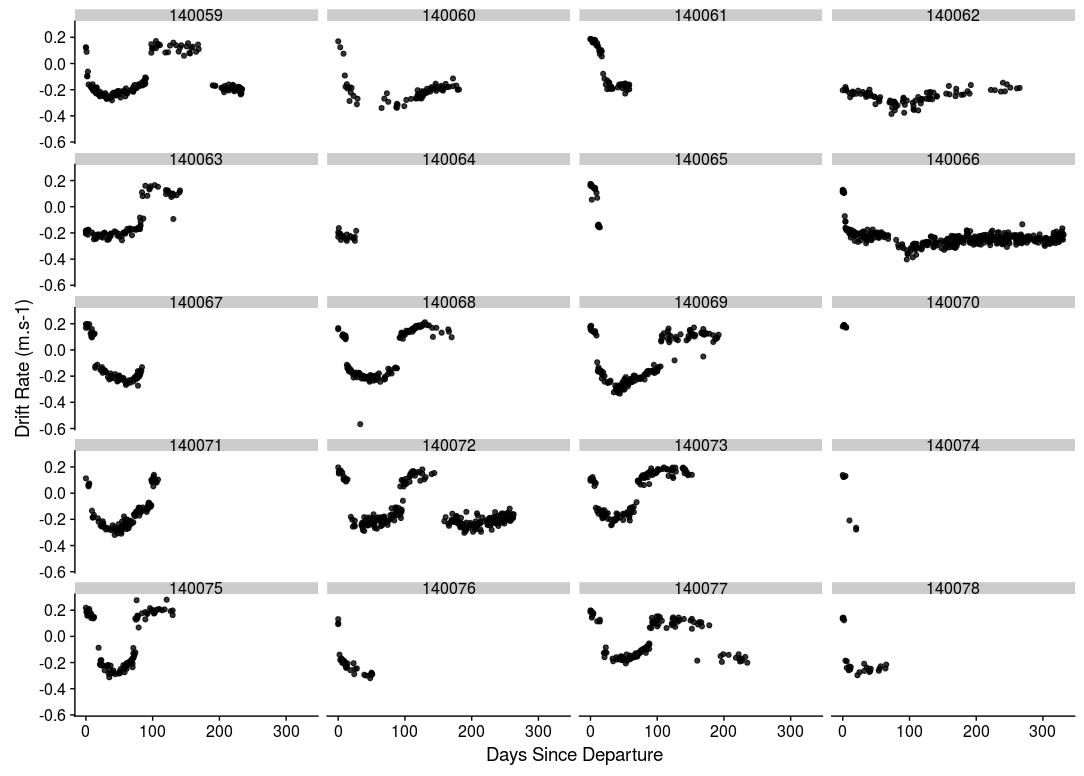


**Figure A7:** Drift rates time series for each juvenile estimated from the vertical speed during drift dives.


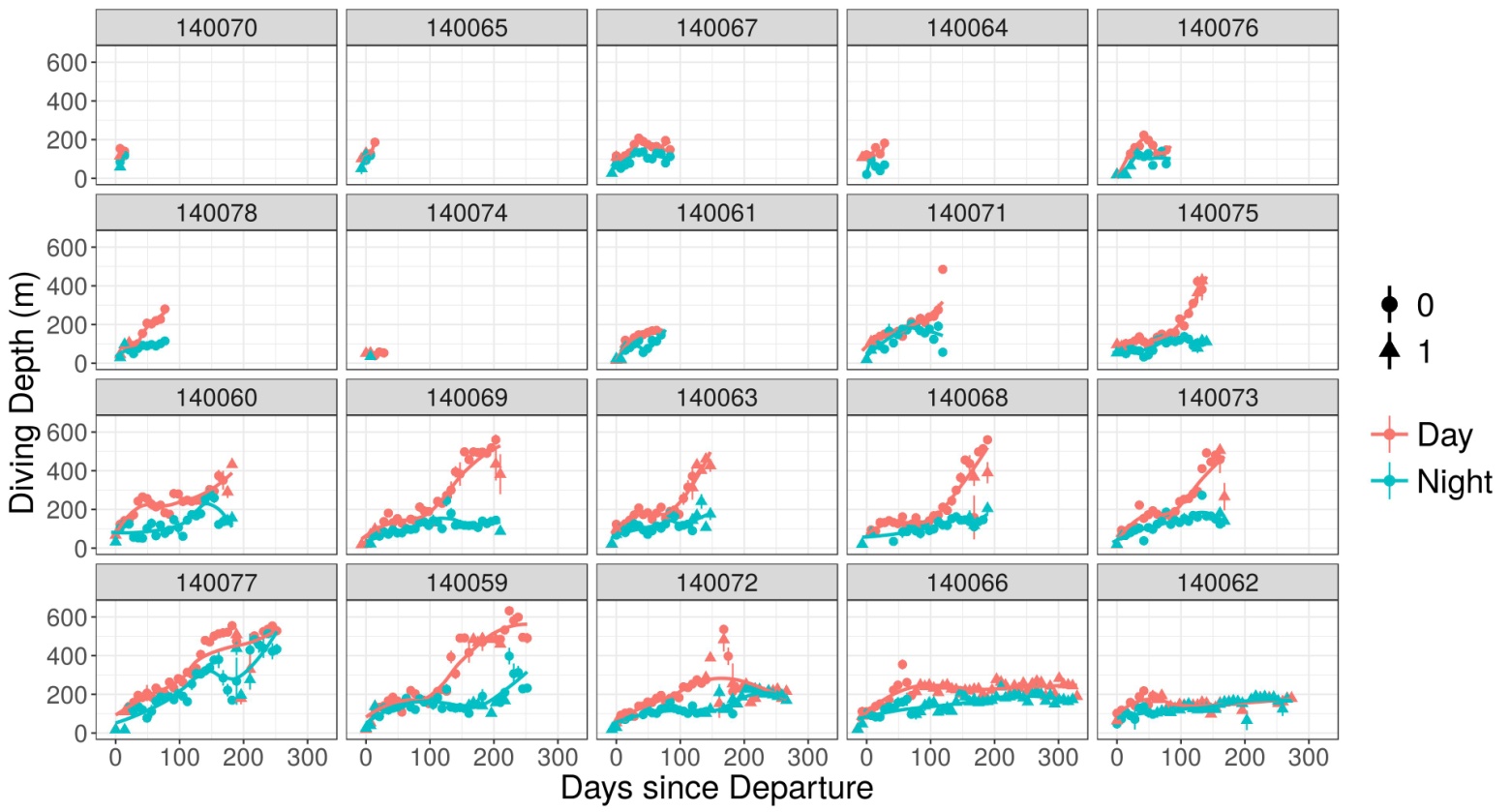


**Figure A8:** Nycthemeral differences between average day and night diving depths over time for each juvenile. Individuals that foraged in the Kerguelen plateau at the end of their trips are 140072, 140066 and 140062. These individuals did not perform demonstrate a strong nycthemeral behaviour as the differences between day and night depths are small.

Tables

**Table A1:** Deployment summaries for the 20 juvenile elephant seals. **BMI:** Body Mass Index at departure (see supplementary material) **On land**: the duration spent on land before departure. **Epi/Benthic Dives:** the proportion of benthic or epi-benthic dives performed on the Kerguelen plateau.

| **Ids** | **Sex** | **Departure**  **Date** | **Departure**  **Mass**  **(kg)** | **Departure**  **Length**  **(cm)** | **BMI** | **Dive**  **Monitoring**  **(days)** | **On-land**  **(days)** | **Number of Dives** | **Epi/benthic**  **Dives (%)** |
| --- | --- | --- | --- | --- | --- | --- | --- | --- | --- |
| 140065 | M | 02/12/2014 | 83.4 | 141 | 3.6 | 19.6 | 2 | 105 | 21 |
| 140070 | M | 17/12/2014 | 98.4 | 150 | 0.8 | 20.8 | 12 | 74 | 41 |
| 140074 | M | 12/12/2014 | 47.2 | 112 | -2.5 | 27.9 | 6 | 109 | 33 |
| 140064 | M | 04/12/2014 | 73.4 | 140 | -3.9 | 38.8 | 0 | 165 | 3 |
| 140061 | M | 21/12/2014 | 97 | 147 | -1.9 | 72.1 | 18 | 399 | 13 |
| 140076 | M | 31/12/2014 | 116.4 | 151 | 5.4 | 82.7 | 25 | 391 | 12 |
| 140078 | F | 20/12/2014 | 76.6 | 145 | -15.6 | 82.7 | 14 | 378 | 14 |
| 140067 | F | 08/12/2014 | 60.6 | 133 | -10.8 | 88.2 | 3 | 539 | 6 |
| 140071 | M | 18/12/2014 | 92.4 | 148 | -3.4 | 122.1 | 13 | 708 | 7 |
| 140075 | F | 11/12/2014 | 59.4 | 123 | -2.2 | 142.4 | 5 | 979 | 8 |
| 140063 | F | 08/12/2014 | 78 | 142 | -4.2 | 157.3 | 4 | 808 | 20 |
| 140073 | F | 17/12/2014 | 74.2 | 132 | -2.2 | 173.9 | 12 | 968 | 9 |
| 140060 | M | 11/12/2014 | 85 | 140 | 2.0 | 192.1 | 8 | 1201 | 9 |
| 140068 | F | 16/12/2014 | 88.6 | 148 | -5.5 | 196.9 | 11 | 1211 | 9 |
| 140069 | F | 19/12/2014 | 64.8 | 122 | -1.3 | 214.3 | 14 | 1120 | 6 |
| 140059 | F | 20/12/2014 | 88 | 142 | -3.9 | 250.9 | 17 | 1379 | 19 |
| 140077 | F | 26/12/2014 | 109.4 | 149 | 5.6 | 253.1 | 20 | 1030 | 10 |
| 140072 | M | 14/12/2014 | 78.8 | 147 | -11.7 | 274.1 | 8 | 1662 | 40 |
| 140062 | F | 08/12/2014 | 61.6 | 130 | -7.1 | 275.3 | 4 | 913 | 78 |
| 140066 | M | 04/12/2014 | 64 | 139 | -17.1 | 338.1 | 8 | 1895 | 72 |
|  |  | Mean±sd | 79.9±17.7 | 139±11 | -3.8±6.2 | 151.2±96.5 | 10.2±6.6 | 802±529 | 22±21 |

**Table A2:** Generalised additive mixed models (GAMMs) outputs corresponded with the time series of Figure 2 of the main manuscript (juveniles). The response variables depth, descent (Desc) and ascent (Asc), swimming effort (Sweff), and drift rates (DRs) are related to days since departure (daysdep). Individuals (ids) are in random effect.

| **Depth** | **Parametric coefficients:** | **Estimate** | **SE** | **t** | **p-value** |  | **Dev.Expl** |
| --- | --- | --- | --- | --- | --- | --- | --- |
|  | (Intercept) | 10.587 | 0.243 | 43.56 | <2e-16 | *** | 31.20% |
|  |  |  |  |  |  |  |  |
|  | **Approximate significance of smooth terms:** | **edf** | **Ref.df** | **F** | **p-value** |  |  |
|  | s(daysdep) | 3.992 | 4 | 1097.25 | <2e-16 | *** |  |
|  | s(ids) | 18.81 | 19 | 78.81 | <2e-16 | *** |  |
|  |  |  |  |  |  |  |  |
| **Desc Sweff** | **Parametric coefficients:** | **Estimate** | **SE** | **t** | **p-value** |  | **Dev.Expl** |
|  | (Intercept) | 6.7186 | 0.1994 | 33.69 | <2e-16 | *** | 28.30% |
|  |  |  |  |  |  |  |  |
|  | **Approximate significance of smooth terms:** | **edf** | **Ref.df** | **F** | **p-value** |  |  |
|  | s(daysdep) | 3.992 | 4 | 440.4 | <2e-16 | *** |  |
|  | s(ids) | 18.34 | 19 | 51.48 | <2e-16 | *** |  |
|  |  |  |  |  |  |  |  |
| **Asc Sweff** | **Parametric coefficients:** | **Estimate** | **SE** | **t** | **p-value** |  | **Dev.Expl** |
|  | (Intercept) | 10.3776 | 0.4552 | 22.8 | <2e-16 | *** | 52.10% |
|  |  |  |  |  |  |  |  |
|  | **Approximate significance of smooth terms:** | **edf** | **Ref.df** | **F** | **p-value** |  |  |
|  | s(daysdep) | 3.998 | 4 | 784.5 | <2e-16 | *** |  |
|  | s(ids) | 18.762 | 19 | 189.8 | <2e-16 | *** |  |
|  |  |  |  |  |  |  |  |
| **DRs** | **Parametric coefficients:** | **Estimate** | **SE** | **t** | **p-value** |  | **Dev.Expl** |
|  | (Intercept) | -0.0576 | 0.02298 | -2.507 | 0.0123 | * | 59.90% |
|  |  |  |  |  |  |  |  |
|  | **Approximate significance of smooth terms:** | **edf** | **Ref.df** | **F** | **p-value** |  |  |
|  | s(daysdep) | 3.967 | 3.999 | 39.284 | <2e-16 | *** |  |
|  | s(ids) | 14.212 | 19 | 4.587 | 1.61E-14 | *** |  |

**Table A3:** Generalised additive mixed models outputs correspond with the time series of

Figure 3 of the main manuscript (adults). Depth, descending swimming effort (Desc Sweff), ascending swimming effort (Asc Sweff), and drift rates (DRs) are related to days since departure (daysdep). Individuals (ids) are in random effect.

| **Depth** | **Parametric coefficients:** | **Estimate** | **SE** | **t** | **p-value** |  | **Dev.Expl** |
| --- | --- | --- | --- | --- | --- | --- | --- |
|  | (Intercept) | 116.361 | 4.863 | 23.93 | <2e-16 | *** | 14.90% |
|  |  |  |  |  |  |  |  |
|  | **Approximate significance of smooth terms:** | **edf** | **Ref.df** | **F** | **p-value** |  |  |
|  | s(daysdep) | 3.98 | 4 | 164.7 | <2e-16 | *** |  |
|  | s(ids) | 7.99 | 8 | 850.7 | <2e-16 | *** |  |
|  |  |  |  |  |  |  |  |
| **Desc Sweff** | **Parametric coefficients:** | **Estimate** | **SE** | **t** | **p-value** |  | **Dev.Expl** |
|  | (Intercept) | 2.473 | 0.376 | 6.575 | 4.92E-11 | *** | 81.40% |
|  |  |  |  |  |  |  |  |
|  | **Approximate significance of smooth terms:** | **edf** | **Ref.df** | **F** | **p-value** |  |  |
|  | s(daysdep) | 3.986 | 4 | 286.9 | <2e-16 | *** |  |
|  | s(ids) | 7.999 | 8 | 7440.8 | <2e-16 | *** |  |
|  |  |  |  |  |  |  |  |
| **Asc Sweff** | **Parametric coefficients:** | **Estimate** | **SE** | **t** | **p-value** |  | **Dev.Expl** |
|  | (Intercept) | 8.8791 | 0.9608 | 9.241 | <2e-16 | *** | 92.20% |
|  |  |  |  |  |  |  |  |
|  | **Approximate significance of smooth terms:** | **edf** | **Ref.df** | **F** | **p-value** |  |  |
|  | s(daysdep) | 3.953 | 3.999 | 3796 | <2e-16 | *** |  |
|  | s(ids) | 8 | 8 | 21524 | <2e-16 | *** |  |
|  |  |  |  |  |  |  |  |
| **DRs** | **Parametric coefficients:** | **Estimate** | **SE** | **t** | **p-value** |  | **Dev.Expl** |
|  | (Intercept) | -0.33933 | 0.00864 | -39.26 | <2e-16 | *** | 55.60% |
|  |  |  |  |  |  |  |  |
|  | **Approximate significance of smooth terms:** | **edf** | **Ref.df** | **F** | **p-value** |  |  |
|  | s(daysdep) | 3.356 | 3.774 | 206.58 | <2e-16 | *** |  |
|  | s(ids) | 7.633 | 8 | 27.43 | <2e-16 | *** |  |

**Table A4:** Generalised additive mixed models outputs corresponded with the time series of Figure 5 of the main manuscript. Prey catch attempt rates (PrCA) are related to days since departure (daysdep). A comparison of the two life stages is included: adult post-breeding females (stadads_pb) and juveniles (stadjuvs). Individuals (ids) are in random effect.

| **PrCA** | **Parametric coefficients:** | **Estimate** | **SE** | **t** | **p-value** |  | **Dev.Expl** |
| --- | --- | --- | --- | --- | --- | --- | --- |
|  | (Intercept) | -1.38018 | 0.01 | -143.5 | <2e-16 | *** | 12% |
|  |  |  |  |  |  |  |  |
|  | **Approximate significance of smooth terms:** | **edf** | **Ref.df** | **F** | **p-value** |  |  |
|  | s(daysdep):stadads_pb | 3.872 | 3.945 | 379.25 | <2e-16 | *** |  |
|  | s(daysdep):stadjuvs | 3.984 | 4 | 433.75 | <2e-16 | *** |  |
|  | s(ids) | 26.186 | 28 | 57.54 | <2e-16 | *** |  |

| **Dive Duration** | **Parametric coefficients:** | **Estimate** | **SE** | **t** | **p-value** |  | **Dev.Expl** |
| --- | --- | --- | --- | --- | --- | --- | --- |
|  | (Intercept) | 44.631 | 1.232 | 36.23 | <2e-16 | *** | 67.30% |
|  | **Approximate significance of smooth terms:** | **edf** | **Ref.df** | **F** | **p-value** |  |  |
|  | s(div.dur):stadads_pb | 1.997 | 2 | 2162.7 | <2e-16 | *** |  |
|  | s(div.dur):stadjuvs | 1.998 | 2 | 2136.3 | <2e-16 | *** |  |
|  | s(ids) | 27.822 | 28 | 408.7 | <2e-16 | *** |  |
| **Tot Sweff** | **Parametric coefficients:** | **Estimate** | **SE** | **t** | **p-value** |  | **Dev.Expl** |
|  | (Intercept) | 54.076 | 1.685 | 32.09 | <2e-16 | *** | 66.10% |
|  | **Approximate significance of smooth terms:** | **edf** | **Ref.df** | **F** | **p-value** |  |  |
|  | s(tot.sweff):stadads_pb | 1.985 | 2 | 2776 | <2e-16 | *** |  |
|  | s(tot.sweff):stadjuvs | 1.998 | 2 | 1286.2 | <2e-16 | *** |  |
|  | s(ids) | 27.823 | 28 | 379.5 | <2e-16 | *** |  |
| **Depth** | **Parametric coefficients:** | **Estimate** | **SE** | **t** | **p-value** |  | **Dev.Expl** |
|  | (Intercept) | 67.754 | 1.962 | 34.53 | <2e-16 | *** | 70.90% |
|  | **Approximate significance of smooth terms:** | **edf** | **Ref.df** | **F** | **p-value** |  |  |
|  | s(depth):stadads_pb | 1.995 | 2 | 4811.2 | <2e-16 | *** |  |
|  | s(depth):stadjuvs | 1.983 | 2 | 2645.9 | <2e-16 | *** |  |
|  | s(ids) | 27.841 | 28 | 259.3 | <2e-16 | *** |  |
| **Bottom PrCA** | **Parametric coefficients:** | **Estimate** | **SE** | **t** | **p-value** |  | **Dev.Expl** |
|  | (Intercept) | 50.031 | 2.591 | 19.31 | <2e-16 | *** | 58.30% |
|  | **Approximate significance of smooth terms:** | **edf** | **Ref.df** | **F** | **p-value** |  |  |
|  | s(bott.tipca):stadads_pb | 1.902 | 1.99 | 68.91 | <2e-16 | *** |  |
|  | s(bott.tipca):stadjuvs | 1.986 | 2 | 68.66 | <2e-16 | *** |  |
|  | s(ids) | 27.889 | 28 | 826.53 | <2e-16 | *** |  |

**Table A5**: GAMMs outputs corresponded to Figure 6 of the main manuscript. The response variable is surface interval in relation to dive duration (div.dur), total swimming effort (tot.sweff), depth, and prey catch attempts (bott.tipca).

**Table A6:** Summary of parameters measured for each individual (ids): initial mass (mi, in kg), departure mass (md, in kg), length at weaning (lg, in cm), time spent onland before departure (onland, in days), scaled mass index (smi), body mass index (bmi), changes in drift rate the first 50 days (slope.dr, in m.s-^1^.day^-1^), first drift rate observed (first.dr, in m.s-1), changes in dive duration the first 50 days (slope.dur, in sec.day-1) and first dive duration (inter.dur, in sec)

| ids | mi | md | lg | onland | sex | smi | bmi.departure | bmi.equipment | slope.dr | first.dr | slope.dur | inter.dur |
| --- | --- | --- | --- | --- | --- | --- | --- | --- | --- | --- | --- | --- |
| 140059 | 88.0 | 75.7 | 142.0 | 17.0 | F | 59.8 | -3.9 | 4.0 | 0.0 | 0.1 | 2.8 | 358.3 |
| 140060 | 85.0 | 79.3 | 140.0 | 8.0 | M | 65.6 | 2.0 | 3.8 | 0.0 | 0.2 | 2.4 | 421.5 |
| 140061 | 97.0 | 83.2 | 147.0 | 18.0 | M | 59.0 | -1.9 | 5.9 | 0.0 | 0.2 | 2.6 | 383.0 |
| 140062 | 61.6 | 59.2 | 130.0 | 4.0 | F | 61.8 | -7.1 | -5.5 | 0.0 | -0.2 | 3.7 | 338.9 |
| 140063 | 78.0 | 75.3 | 142.0 | 4.0 | F | 59.5 | -4.2 | -6.0 | 0.0 | -0.2 | 2.1 | 381.4 |
| 140064 | 73.4 | 73.4 | 140.0 | 0.0 | M | 60.7 | -3.9 | -7.8 | 0.0 | -0.2 | 0.9 | 397.5 |
| 140065 | 83.4 | 82.0 | 141.0 | 2.0 | M | 66.3 | 3.6 | 0.8 | 0.0 | 0.2 | 1.9 | 463.2 |
| 140066 | 64.0 | 59.1 | 139.0 | 8.0 | M | 50.0 | -17.1 | -15.8 | 0.0 | 0.1 | 1.5 | 344.1 |
| 140067 | 60.6 | 58.8 | 133.0 | 3.0 | F | 57.2 | -10.8 | -10.7 | 0.0 | 0.2 | 3.0 | 324.0 |
| 140068 | 88.6 | 80.6 | 148.0 | 11.0 | F | 55.9 | -5.5 | -3.9 | 0.0 | 0.2 | 0.4 | 423.3 |
| 140069 | 64.8 | 56.2 | 122.0 | 14.0 | F | 71.7 | -1.3 | 9.1 | 0.0 | 0.2 | 1.6 | 305.0 |
| 140070 | 98.4 | 89.1 | 150.0 | 12.0 | M | 59.3 | 0.8 | 3.1 | 0.0 | 0.2 | 6.9 | 424.5 |
| 140071 | 92.4 | 82.7 | 148.0 | 13.0 | M | 57.4 | -3.4 | -0.1 | 0.0 | 0.1 | 1.3 | 391.9 |
| 140072 | 78.8 | 73.3 | 147.0 | 8.0 | M | 52.0 | -11.7 | -12.3 | 0.0 | 0.2 | 0.6 | 337.8 |
| 140073 | 74.2 | 66.3 | 132.0 | 12.0 | F | 66.0 | -2.2 | 4.3 | 0.0 | 0.1 | 3.1 | 320.8 |
| 140074 | 47.2 | 44.0 | 112.0 | 6.0 | M | 73.5 | -2.5 | 5.6 | 0.0 | 0.1 | -2.0 | 218.8 |
| 140075 | 59.4 | 56.5 | 123.0 | 5.0 | F | 70.2 | -2.2 | 2.2 | 0.0 | 0.2 | 1.0 | 317.9 |
| 140076 | 116.4 | 94.9 | 151.0 | 25.0 | M | 61.8 | 5.4 | 19.6 | 0.0 | 0.1 | 6.0 | 325.8 |
| 140077 | 109.4 | 92.8 | 149.0 | 20.0 | F | 63.1 | 5.6 | 15.5 | 0.0 | 0.2 | 4.2 | 420.5 |
| 140078 | 76.6 | 67.2 | 145.0 | 14.0 | F | 49.7 | -15.6 | -11.7 | 0.0 | 0.1 | 0.4 | 322.8 |

Bivand, R., Lewin-Koh, N., Pebesma, E., Archer, E., Baddeley, A., Bearman, N., … Turner, R. (2017). maptools: Tools for Reading and Handling Spatial Objects (Version 0.9-2). Retrieved from https://cran.r-project.org/web/packages/maptools/index.html

Guinet, C. (1992). Croissance des éléphants de mer de l’archipel Crozet (46° 25’S, 51° 45’E) pendant leur première année de vie. *Mammalia*, *56*(3), 459–468.

Guinet, C., Roux, J. P., Bonnet, M., & Mison, V. (1998). Effect of body size, body mass, and body condition on reproduction of female South African fur seals (Arctocephalus pusillus) in Namibia. *Canadian Journal of Zoology*, *76*(8), 1418–1424. doi:10.1139/z98-082

Hindell, M. A., McConnell, B. J., Fedak, M. A., Slip, D. J., Burton, H. R., Reijnders, P. J., & McMahon, C. R. (1999). Environmental and phsysiological determinants of successful foraging by naive southern elephant seal pups during their first trip to sea. *Canadian Journal of Zoology*, *77*(11), 1807–1821. doi:10.1139/z99-154

Hindell, M. A., Slip, D. J., & Burton, H. R. (1991). The Diving Behavior of Adult Male and Female Southern Elephant Seals, Mirounga-Leonina (Pinnipedia, Phocidae). *Australian Journal of Zoology*, *39*(5), 595–619. doi:10.1071/zo9910595

Irvine, L. G., Hindell, M. A., van den Hoff, J., & Burton, H. R. (2000). The influence of body size on dive duration of underyearling southern elephant seals (Mirounga leonina). *Journal of Zoology*, *251*(4), 463–471. doi:10.1111/j.1469-7998.2000.tb00802.x

Le Boeuf, B. J., Morris, P. A., Blackwell, S. B., Crocker, D. E., & Costa, D. P. (1996). Diving behavior of juvenile northern elephant seals. *Canadian Journal of Zoology*, *74*(9), 1632–1644. doi:10.1139/z96-181

McConnell, B., Fedak, M., Burton, H. R., Engelhard, G. H., & Reijnders, P. J. H. (2002). Movements and foraging areas of naïve, recently weaned southern elephant seal pups. *Journal of Animal Ecology*, *71*(1), 65–78. doi:10.1046/j.0021-8790.2001.00576.x

O’Toole, M., Hindell, M., Charrassin, J.-B., & Guinet, C. (2014). The foraging behaviour of Southern Elephant Seals over the Kerguelen Plateau. *Marine Ecology Progress Series*, *502*, 281–294. doi:10.3354/meps10709
